# Diagnostic potential of a multi-antigen ELISA for feline leishmaniosis

**DOI:** 10.1186/s13071-026-07320-5

**Published:** 2026-03-16

**Authors:** Clara M. Lima, Óscar Felgueiras, Margarida Brito, Mariaelisa Carbonara, Domenico Otranto, Andreia Magalhães, Rui Ferreira, Joana Tavares, Luís Cardoso, Anabela Cordeiro da Silva, Nuno Santarém

**Affiliations:** 1 Host-Parasite Interaction Group, Institute for Research and Innovation in Health (i3S), University of Porto, Porto, Portugal; 2 Microbiology Laboratory, Department of Biological Sciences, Faculty of Pharmacy, University of Porto, Porto, Portugal; 3 Department of Mathematics, Faculty of Sciences and Centre for Mathematics, University of Porto, Porto, Portugal; 4 Department of Veterinary Medicine, University of Bari, Bari, Italy; 5 Department of Veterinary Clinical Sciences, City University of Hong Kong, Hong Kong, China; 6 Animal Blood Bank (BSA), Porto, Portugal; 7 School of Medicine and Biomedical Sciences (ICBAS), University of Porto, Porto, Portugal; 8 Department of Veterinary Sciences, and Animal and Veterinary Research Centre (CECAV), University of Trás-os-Montes e Alto Douro (UTAD), Vila Real, Portugal; 9 Associate Laboratory for Animal and Veterinary Sciences (AL4AnimalS), Lisboa, Portugal

**Keywords:** DAT, Feline leishmaniosis, IFAT, LicTXPNx-ELISA, PCA, RK28-ELISA, RK39-ELISA, RKDDR-ELISA, SPLA-ELISA

## Abstract

**Background:**

*Leishmania infantum* is a sand fly-transmitted zoonotic protozoan, endemic in the Mediterranean basin and responsible for human, canine (CanL), and feline (FeL) leishmaniosis. While dogs are the primary reservoir host, a growing number of FeL cases have been reported in this region despite the absence of pathognomonic clinical signs and limited diagnostic tools. Herein, we evaluate the performance of seven serological tools for CanL in detecting antibodies to *Leishmania* in cats, aiming to improve FeL diagnosis.

**Methods:**

Five ELISAs based on *Leishmania*-specific antigens (soluble promastigote *Leishmania* antigens, SPLA; recombinant *Leishmania* proteins K39 [rK39], K28, and KDDR, and *L. infantum* cytosolic peroxiredoxin, LicTXNPx), indirect fluorescent antibody test (IFAT), and direct agglutination test (DAT) were compared for detecting anti-*Leishmania* antibodies in 274 cats. Blood samples from the same cats were molecularly tested. Statistical analysis was performed based on clustering of multivariate serological data. Reference serological profiles were first defined in a control group. Study group data were subsequently classified according to these profiles, with principal component analysis used for dimensionality reduction and graphical representation. Associations between seropositivity and clinicopathological alterations were determined using seropositivity thresholds.

**Results:**

Cats exhibited attenuated and heterogeneous antibody responses to *L. infantum* serological tests. Agreement between individual tests was variable, with poor concordance when single markers were considered. Multivariate analysis, based on clustering of serological responses, showed that positivity to multiple antigens was associated with clinically affected cats. Positivity to multiple *Leishmania*-specific ELISA antigens was associated with diverse clinical presentations and prognostic laboratory alterations, including anaemia, thrombocytopenia, and hypergammaglobulinaemia.

**Conclusions:**

Integrating multi-antigen ELISA, particularly rK39, SPLA, and LicTXNPx, into FeL diagnostic workflows, alongside molecular and clinical assessment, improves epidemiological surveillance, early detection, and disease management. These findings support the development of serological strategies tailored to feline hosts for enhanced surveillance and management.

**Graphical Abstract:**

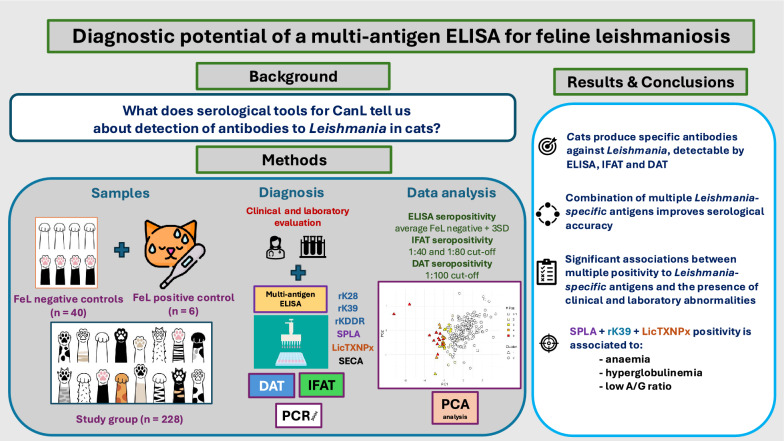

**Supplementary Information:**

The online version contains supplementary material available at 10.1186/s13071-026-07320-5.

## Background

Leishmaniosis caused by *Leishmania infantum* is a protozoan vector-borne zoonosis of global veterinary and public health significance. Given the zoonotic nature of the disease, research on primary reservoir hosts is highly relevant for human and animal health. Dogs have long been at the centre of research on host-parasite interactions, clinical investigations, drug development, and epidemiology of zoonotic leishmaniosis caused by *L. infantum*. This is supported by not only the high burden of the disease on dogs but also their established role as the main reservoirs of *L. infantum*. Therefore, control of canine leishmaniosis (CanL) remains a cornerstone of public health and veterinary interventions to reduce parasite transmission [1–3]. However, the dog is not the only companion animal that can be targeted by these parasites. The first documented case of *L. infantum* infection in a cat was reported in 1912. Still, the epidemiological importance and veterinary relevance of feline leishmaniosis (FeL) have received limited attention until recent decades [2]. The reason for this apparent neglect for most of the past century was the limited number of clinical cases of FeL compared to CanL [4]. However, xenodiagnostic evidence of the infectiousness of *L. infantum*-infected cats to sand fly vectors [5, 6], followed by the demonstration of metacyclic parasite transmission from FeL-fed phlebotomines to vertebrate hosts [7], provided insights into feline reservoir competence toward *L. infantum*. Although cats are recognized as established reservoirs of *Leishmania*, their role in sustaining the *L. infantum* life cycle in the absence of canine hosts remains to be thoroughly evaluated. Interestingly, in regions where both dogs and cats occur in sympatry, the overall seroprevalence of FeL is often reported to be lower than that of CanL [8–12]. Nonetheless, a recent multicentre study from Portugal, Spain, southern France, Italy, Greece, and Israel estimated 17.3% global seroprevalence of feline exposure to *L. infantum* in these Mediterranean countries [13]. Importantly, both epidemiological and clinical reports of leishmaniosis in wild and captive felids in this region also support the natural susceptibility of other felid species to *Leishmania* infection [14, 15].

The clinical and laboratory findings associated with FeL often overlap with those of CanL. Infected cats can present with lymphadenopathy, fever, weight loss, dermatological lesions, ocular signs, and involvement of mucocutaneous transitions and visceral organs. Atypical and sporadic presentations of granulomatous rhinitis [16], nodular dermatitis [17], and granulomatous enteritis [18] have also been associated with FeL. The most reported clinicopathological alterations include azotaemia, non-regenerative normocytic-normochromic anaemia, hyperglobulinaemia, and hyperproteinaemia [19], often accompanied by increased acute-phase pro-inflammatory proteins and mono- or polyclonal gammopathy [20].

Limited understanding of feline immunopathology continues to constrain in-depth characterization of its immune mechanisms—particularly the extent and role of adaptive immune response upon feline infection with *Leishmania*. So far, it has been shown that cats from leishmaniosis-endemic regions develop increased interferon-gamma production and low levels of specific antibodies against the parasite [21]. Overall, it seems that, like in dogs, cellular immune responses play an effective role in managing feline infection's advance to disease, the latter being characterized by detectable antibodies through serodiagnosis [22–24]. Compared to dogs, there is general agreement that cats produce lower levels of *Leishmania*-specific antibodies [2, 12, 19].

Still, the presence of antibodies in the context of FeL has enabled their use in disease management and diagnosis. In fact, even in prior indirect fluorescent antibody test (IFAT) validation for FeL diagnosis, with a cut-off titre of 80 [44], this tool was widely applied in serological surveys [10, 11, 26–35] and has been preferentially chosen over other serological methods for FeL clinical diagnosis [20]. Despite its widespread use, IFAT is limited by subjective interpretation of fluorescence, lack of standardization across laboratories, and known issues with cross-reactivity with other pathogens. In fact, the impact of antibody cross-reactivity, extensively reported for CanL, is mostly unknown in FeL. Other serological approaches such as the direct agglutination test (DAT) [9, 35], western blotting [36], and enzyme-linked immunosorbent assays (ELISA) [8, 37, 38], have been used to a lesser extent. Discordant results between seropositivity to different serological and molecular approaches have been documented [36, 39], also considering that multiple serological assays have been simultaneously employed in a few studies [13, 39]. Importantly, despite the increasing relevance of *L. infantum* infection in cats, serological approaches to its diagnosis remain poorly standardized. Current diagnostic methods show variable sensitivity (Sn) and specificity (Sp) and often lack agreement with each other, and with direct parasitological techniques. Available research on the differential performance of serological assays for detecting anti-*Leishmania* immunoglobulin G (IgG) in felines remains limited, thereby restricting veterinary diagnostic laboratories from offering a wider range of diagnostic solutions. Moreover, comparison of multiple antigenic targets or recombinant proteins for FeL diagnosis have not been considered, despite evidence that such comparisons improve CanL diagnosis accuracy [40]. Additionally, there is a lack of comprehensive data regarding the performance of serological assays in both clinical and subclinical infections, and the predictive value of a positive test result in cats with diverse clinical backgrounds remains uncertain. The lack of comparative data hampers the development of feline-specific diagnostic algorithms and may lead to underdiagnosis or misinterpretation of serological data, with clinical and epidemiological consequences [4]. Considering the limitations of FeL serological evaluation, it is necessary to bring FeL serological assessment into the twenty-first century and provide veterinarians, epidemiologists, and researchers with information on the usefulness of these tools. To contribute to this goal, we adopted a comparative approach to assess potential of classic serological assays for CanL diagnosis in detecting anti-*Leishmania* antibodies in cats. We used an in-house ELISA based on five different *Leishmania*-specific antigens (soluble promastigote *Leishmania* antigen, SPLA; *Leishmania*-specific recombinant proteins K39, K28, and KDDR; and *L. infantum* cytosolic peroxiredoxin, LicTXNPx) and a non-*Leishmania*-related antigen (soluble *Escherichia coli* antigen, SECA) and compared the seropositivity readout with IFAT, DAT, and polymerase chain reaction (PCR). The generated serological data were also interpreted by means of a principal component analysis (PCA) to stratify serological responses. Serological findings were further framed alongside clinical and clinicopathological observations to pinpoint meaningful test combinations that offer the best FeL predictive diagnostic values.

## Methods

### Feline serum samples

A total of 274 feline serum or plasma and 168 whole-blood samples were included in the study. All samples were stored at − 20 ºC until analysis. Samples were organized into three groups: Group 1 (FeL −): 40 negative control cats from Madeira Island, a geographical area where leishmaniosis is not endemic. These samples had been previously tested seronegative by IFAT and DAT. Group 2 (FeL +): Positive control samples were obtained from six FeL clinical cases (designated as A, B, C, D, E, and F) with confirmed diagnosis by either direct (parasitological) and/or indirect (serological) tests, and positive therapeutic outcome in five of the six animals (Additional file 1: Table S1). Group 3 (study group): 228 feline serum or plasma samples obtained between the years 2021 and 2024 from cats living in CanL-endemic regions of Portugal. Approximately 1 ml of peripheral blood was collected under mild sedation from either jugular or cephalic venepuncture and transferred to a serum (dry tube) or plasma (k3 EDTA) tube. Sampled animals included stray cats admitted for trap-neuter-return programmes or others available for adoption at municipal animal shelters. Animals < 6 months old were not included in the study. Cats were submitted to a general clinical examination that included signalment (i.e. age, sex, and breed), body condition score (I, cachectic; II, underweight; III, regular; IV, overweight; V, obese), and presence of external parasites. Cats were classified as apparently healthy or clinically sick based on the identification of dermatological lesions (e.g. alopecia, crusts, ulcers, dermatitis, nodules, onychogryphosis, papules, wounds); gastrointestinal signs (e.g. diarrhoea, vomit); neurological alterations (e.g. ataxia; vestibular syndrome; head tilts; nystagmus); oral lesions (e.g. gingivitis, tonsilitis, mucositis, ulcers, periodontal disease); ophthalmic lesions (e.g. blepharitis, conjunctivitis, corneal lesions, ocular discharge, periocular alopecia; uveitis, retinitis); respiratory signs (e.g. dyspnoea, nasal discharge, rhinitis); systemic signs (e.g. dehydration, emaciation or cachexia, fever, lymphadenopathy, pale mucous membranes, jaundice); and urinary signs (e.g. dysuria, haematuria, pyuria).

### Serological survey

All samples were tested for *Leishmania*-specific antibodies using seven different serological techniques, including five *Leishmania*-specific ELISA antigens; IFAT (cut-offs of 1:40 and 1:80) and DAT (cut-off of 1:100). The Sp of ELISA serological response to *Leishmania* antigens was addressed by a non- *Leishmania*-related antigen.

#### ELISA

Six ELISA antigens were used, including five *Leishmania*-specific antigens: SPLA; recombinant *L. infantum* kinesins 39 [rK39], 28 [rK28], and degenerated derived repeats [rKDDR]; LicTXNPx; and SECA. For SPLA, *L. infantum* promastigotes were obtained as previously described using *L. infantum* MHOM/MA/67/ITMAP-263 [41]. The SECA was produced in-house according to C(S) Lima et al. [42]. The recombinant protein LicTXNPx was produced in-house as described by Castro et al. [43]. Lyophilized rK39 and rK28 antigens were provided by Dr. Steven Reed (Infectious Disease Research Institute, Seattle, WA, USA) and suspended in 0.22 μm deionized membrane-filtered water, quantified and stored in single-use aliquots at − 20 ºC until use. The recombinant protein kDDR was provided by Dr. Ricardo Fujiwara (Universidade Federal de Minas Gerais, Belo Horizonte, Brasil) and stored at − 20 °C in single aliquots until use.

##### ELISA protocol

The canine ELISA protocol previously described for the detection of anti-*Leishmania* IgG [41] was adjusted to detect anti-*Leishmania* IgG in feline serum or plasma samples. High-binding 96-well flat-bottom polystyrene plates (Greiner Bio-One, Kremsmünster, Austria) were coated in triplicate for each antigen with 50 µl/well of 10 µg ml^−1^ SPLA and SECA and 5 µg ml^−1^ rK39, rK28, rKDDR, and LicTXNPx. Antigen dilutions were performed in 0.05 M carbonate-bicarbonate buffer (pH 9.6). Following 24 h incubation at 4 °C, the antigen solution was discarded and plates were blocked with 200 μl of a 3% dry milk suspension (Molico dry skimmed milk, Nestlé, Switzerland) diluted in phosphate-buffered saline (PBS). Plates were incubated for 30 min at 37 °C and washed three times with a 0.05% solution of PBS-Tween 20 (Sigma-Aldrich, Steinheim, Germany), using an automated ELISA plate washer (BioTek, Winooski, VT, USA). Diluted samples (1:800 in PBS-0.05% Tween 20) were then added at 100 μl/well for each antigen and incubated for 30 min at 37 °C. A washing step was repeated and 100 μl/well of rabbit HRP-conjugated anti-cat IgG (anti-cat IgG [H + L]-peroxidase antibody produced in rabbit, SAB3700080; Sigma-Aldrich, Saint Louis, MO, USA) diluted 1:6000 in PBS-0.05% Tween 20 was added to each well. Following 30 min of incubation at 37 °C, plates were washed as described above. Detection was performed with 100 μl/well of 0.5 mg/ml o-phenylenediamine dihydrochloride (OPD; Sigma-Aldrich, St. Louis, MO, USA) diluted in citrate buffer (pH 5.2) with 1 μl/ml of H_2_O_2_ (30%). After 10 min incubation at room temperature (RT) in the dark, the reaction was stopped with 50 μl/well of 3 M hydrochloric acid (HCl). Absorbance was read at 490 nm wavelength in an automated spectrophotometer (Synergy 2; Agilent, Winooski, VT, USA). Results were registered as optical densities (OD) by BioTek Gen5 Microplate Reader and Imaging Software (Agilent, Winooski, VT, USA). Plate analysis was performed using an Excel (Microsoft, Redmond, WA, USA) spreadsheet. For each antigen, the final ELISA result was calculated as the average of technical triplicates from at least two independent assays, subtracting the average OD value of the respective blanks. Serum from a cat confirmed to be infected with *L. infantum* and diseased was used as a positive control on each ELISA plate.

#### IFAT

The IFAT protocol validated for FeL by Iatta et al. [25] was followed with minor modifications. The same *L. infantum* laboratory strain used for SPLA production (MHOM/MA/67/ITMAP-263) was expanded during 7 days in a T75 flask containing complete RPMI until stationary metacyclic *L. infantum* promastigotes were obtained. Promastigotes were then washed with PBS and centrifuged (1800×*g*, 10 min, at RT) three times. Next, promastigotes were resuspended in PBS to a final concentration of 5 × 10^6^ mL^−1^. Then, 40 µl of the suspension was distributed into each well of the diagnostic slide (Multitest 12-well slide, 0.7-cm well diameter, MP Biomedicals, Eschwege, Germany). Slides were allowed to air-dry at 37 °C and then acetone-fixed for 2 h at − 20 °C. After acetone had evaporated, the air-dried slides were preserved at − 20 °C until use.

Serum or plasma samples were diluted 1:40 in PBS and 40 µl of the dilution was placed in the centre of the designated well. A positive and negative control were included in each slide, together with a blank. Next, slides were incubated for 30 min at 37 °C inside a humidified chamber. After sample incubation, slides were rinsed with PBS, followed by two independent PBS washess of 5 min each, then rinsed in distilled water. After washing, slides were allowed to dry for at least 10 min at 37 °C. Then, 40 µl of goat anti-cat IgG-fluorescein isothiocyanate conjugate (Sigma-Aldrich, Saint Louis, MO, USA) diluted 60 times in 0.05% Evans blue was added to each well, and the slides were incubated and washed as described above. A drop of mounting medium was placed on each well before applying a coverslip. Slides were examined under a fluorescence microscope (Nikon Eclipse E400, Feasterville, PA, USA). Positivity was determined whenever a sample produced a clear cytoplasmic membrane and flagellar fluorescence in at least 50% of the observation field, as assessed at 20× and 40× magnification. All samples positive at 1:40 dilution were subsequently tested at 1:80 dilution.

#### DAT

The direct antiglobulin test (DAT) protocol for titration of antibodies to *Leishmania* and seropositivity cut-off value was followed as described by Cardoso et al. [9], using freeze-dried *L. donovani* (MHOM/SD/68/1S) promastigotes as reagent (5 × 10^7^ ml^−1^ resuspended in physiological saline, 0.9% NaCl) (Amsterdam University Medical Centres, Academic Medical Centre at the University of Amsterdam, Department of Medical Microbiology, Section Experimental Parasitology, Amsterdam, The Netherlands). Briefly, serum samples were diluted in saline (0.9% NaCl) containing 1.56% β-mercaptoethanol. For each sample, serial dilutions were prepared in a V-shaped low-binding microtiter plate (Greiner Bio-One, Frickenhausen, Germany), starting at 1:25 and ending at 1:3200 dilution. Following 1 h incubation at 37 °C, 50 μl of the DAT antigen was distributed in each well and the plates were incubated for 18 h (RT). A positive result was determined when agglutination (large diffuse blue mats) was clearly visible after 18 h incubation at RT. Sera from a FeL case (confirmed by rK39-ELISA and parasite culture) were used as the positive control. Sera from a healthy, non-infected, indoor cat (rK39-ELISA negative, IFAT negative, and blood PCR negative) were used as the negative control. The DAT cut-off (100) was established at a serum dilution of 1:100, as previously described [9].

### Molecular survey

DNA was extracted from the peripheral blood of a subgroup of study samples (*n* = 168) and tested by real-time PCR (qPCR) for the detection of *L. infantum* kDNA minicircle (120 bp) using primers, probes, and protocol described elsewhere [25].

### Statistical analysis and graphical representations

Statistical analyses were performed to define seropositivity thresholds, explore associations between serological results and clinical or clinicopathological variables, and characterize multivariate serological profiles. Analyses and graphical representations were performed using GraphPad Prism software version 9 (San Diego, CA, USA), IBM SPSS Statistics for Windows version 27.0 (IBM, Armonk, NY, USA), R version 4.4.3 (R Core Team, 2024) and Microsoft Excel (Microsoft Corp., 2025).

### Performance and serological profiling of the in-house ELISA

To evaluate the capacity of the different antigens used in the in-house ELISA to be detected in the context of FeL, two distinct control cohorts were used: a group of 6 cats diagnosed with FeL (FeL + group; Additional file 1: Table S1) and a group of 40 cats from a non-endemic region (FeL − group). Differences in ELISA seroreactivity against each one of the five *Leishmania*-specific antigens and the non-related antigen SECA were studied using the non-parametric Mann-Whitney U-test, followed by Shapiro-Wilk or Kolmogorov-Smirnov normality tests. Significance of the differences between values (assumed for *P* < 0.05) was determined for the FeL + and FeL − control groups and for the sub-populations of healthy and sick individuals from the study group.

Quantitative OD values were log-transformed and subsequently standardized using *z*-scores. Linear relationships between different combinations of pairs of ELISA antigens were measured using pairwise Pearson correlation. Statistical significance was assessed using adjusted *P*-values, corrected with the Benjamini-Hochberg (BH) method for multiple testing. This analysis was complemented with a dendrogram derived from hierarchical clustering. To explore the data's underlying structure, a PCA was performed. The number of clusters was estimated using the elbow method, and individuals were grouped accordingly using *k*-means clustering. The resulting clusters were visualized in the PCA space, with the variable contributions to the principal components also examined. Subsequently, the study group was analysed. The same pre-processing steps—log transformation followed by standardization—were applied. To ensure comparability, the study group data were projected onto the principal component axes previously obtained from the control group. Clustering was then performed using the centroid method based on the control group cluster definitions. PCA-based visualizations were used to depict the spatial distribution of seropositive results across clusters *k*1 and *k*2 for individual *Leishmania*-specific ELISA antigens, multi-antigen seropositivity, IFAT (cut-offs: 40 and 80) and DAT (cut-off: 100).

### Determination of ELISA cut-off values

Cut-off values for seropositivity to SPLA, rK39, rK28, rKDDR and LicTXNPx were established using FeL-negative control samples (*n* = 40). For each antigen, the cut-off was defined as the mean OD value of the FeL-negative group plus 3*standard deviations (SD) (Additional file 2: Table S2). Samples with values equal to or above the respective cut-off were considered seropositive.

### Associations between *Leishmania*-specific antibodies and the presence of clinical and laboratory manifestations of disease

Associations between seropositivity to the different ELISA antigens, IFAT, DAT and PCR with clinical and clinicopathological laboratory parameters were determined based on a contingency analysis for statistical comparisons (Pearson chi-square test [*χ*2] or Fisher’s exact test [FET]). The analysis was performed following a binomial stratification to describe positivity (1) or negativity (0) to any technique, as determined by the proposed cut-offs. Non-random associations were defined for* P* < 0.05. Agreement beyond chance between results for the same sample was calculated by Cohen’s kappa coefficient (*k*). The strength of agreement was classified as poor if *k* ≤ 0.00; slight if 0.00 ≥ *k* < 0.20; fair for 0.21 ≥ *k* < 0.40; moderate for 0.41 ≥ *k* < 0.60; substantial for 0.61 ≥ *k* < 0.80 and almost perfect for 0.81 ≥ *k* < 1.00 [45].

Associations between diagnostic test and health status were determined for the study group (*n* $=$ 228), where animals were divided in two subgroups: sick (*n* $=$ 71; cats presenting at least one clinical sign of disease) versus apparently healthy (*n* $=$ 157; cats with no recognizable clinical signs of disease during physical examination). Associations between being seropositive and presenting specific clinical signs on physical examination (e.g. dermatological lesions [e.g. alopecia, crusts, dermatitis, dry seborrhoea, erythema, furfuraceous, hyperkeratosis, nodules, ulcers, wounds]; gastrointestinal signs [e.g. vomiting, diarrhoea]; neurological signs [e.g., ataxia, head tilt, Horner syndrome]; oral lesions [e.g. periodontal disease, ulcers, gingivitis]; ophthalmic lesions [e.g. conjunctivitis, corneal lesions, epiphora, nodular blepharitis, uveitis]; respiratory signs [e.g. nasal discharge, nasal occlusion, inspiratory stertor, dyspnoea]; systemic [e.g. cachexia/underweight, dehydration, dull coat, fever, lethargy, lymphadenopathy, pale mucous membranes, jaundice, weight loss] and urinary signs [e.g. cystitis, dysuria, haematuria, kidney disease, lower urinary tract infections]) or clinicopathological laboratory findings (including haematological [*n* = 87] and serum biochemistry parameters [*n* = 86]) were also calculated. Haematological parameters were analysed and categorized into high or low red blood cell count (RBC); high or low haematocrit (Ht); high or low haemoglobin; high or low mean cell haemoglobin concentration; high or low mean cell volume; high or low red blood distribution width; leucocytosis, leukopenia, neutrophilia, neutropenia, lymphocytosis, lymphopenia, monocytosis, eosinophilia, basophilia, thrombocytosis and thrombocytopenia. Serum biochemistry parameters included hypoalbuminaemia, hyperalbuminaemia, hypoglobulinaemia, hyperglobulinaemia, hypoproteinaemia, hyperproteinaemia, high or low albumin/globulin ratio (A/G); high alanine aminotransferase (ALT), high creatinine (Crea) and high blood urea nitrogen (BUN). Blood cell counts and serum biochemistry were performed by three different veterinary diagnostic laboratories in Portugal. Abnormal readings for each parameter were determined according to the laboratory reference intervals for each test.

## Results

### Performance of the in-house ELISA in detecting *Leishmania*-specific antibodies

**Fig. 1:**
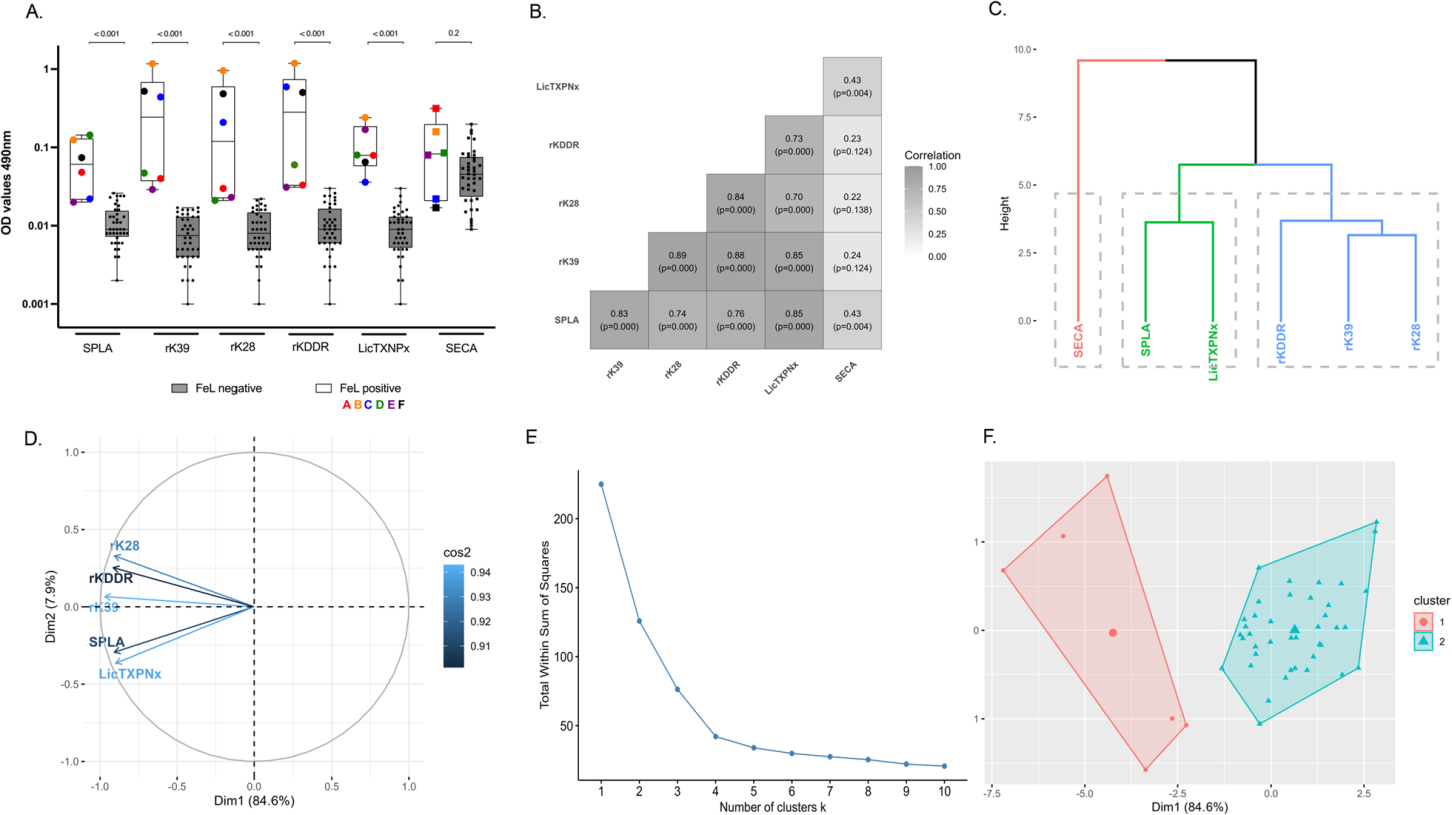
Seroreactivity relationships between feline leishmaniosis-positive (FeL +) and -negative (FeL −) control groups against *Leishmania*-specific and non-specific antigens. **A** Distribution of IgG seroreactivity against SPLA, rK39, rK28, rKDDR, LicTXNPx and SECA by ELISA in FeL + (individually represented by a coloured dot) and FeL − (individually represented by a black dot) controls. Each dot represents the average of at least two independent assays, each performed in technical triplicate for each sample. The Y-axis represents optical density (OD) values measured at 490 nm, plotted on a log10 scale. Differences in median values for the same antigen were assessed using the Mann-Whitney U test. **B** Pearson correlation between the different serological responses observed against *Leishmania*-specific ELISA antigens SPLA, rK39, rK28, rKDDR and LicTXNPx and to the non-related antigen SECA. **C** Dendrogram of hierarchical relations between *Leishmania*-specific serological responses against each ELISA antigen. **D** Two-dimensional (2D) representation of the principal component analysis (PCA) space and contribution of each antigen—represented by independent vectors—to the generated principal components (PC). **E** Partition of the control data set into k-clusters, estimated by the elbow method. **F** Partition of the control data set into two *k*-clusters. ELISA, enzyme-linked immunosorbent assay; LicTXNPX, *Leishmania infantum* recombinant cytosolic peroxiredoxin protein; OD, optical densities; rK28, *L. infantum* recombinant kinesin 28; rK39, *L. infantum* recombinant kinesin 39; rKDDR, *L. infantum* recombinant kinesin degenerated derived repeat; SPLA, soluble promastigote *Leishmania* antigens

Although absolute OD values varied among different antigens in the FeL + group, all five *Leishmania*-specific ELISA antigens (SPLA, rK39, rK28, rKDDR, LicTXNPx) presented significantly higher seroreactivity (*P* < 0.001) in FeL + than in FeL − controls (Fig. 1A). In contrast, the non-related antigen SECA, showed no significant differences (*P* = 0.2) between groups. Within the FeL + group, cats A and D (also FIV+) exhibited higher seroreactivity to SPLA and LicTXNPx than to rK39, rK28, and rKDDR. On the other hand, controls B, C and F (FIV−) exhibited greater seroreactivity to rK39, rK28 and rKDDR than to SPLA and LicTXNPx (Fig. 1A; Additional file 2: Table S2).

The FeL + group was further analysed to characterize *Leishmania*-specific seroreactivity associated with each one of the five ELISA antigens (Fig. 1). To evaluate whether these antigens might complement each other in their recognition, correlation analysis (Fig. 1B) and hierarchical clustering (Fig. 1C) were performed. The unrelated antigen SECA was included to address non-*Leishmania* seroreactivity. There was a strong correlation between seroreactivity to all *Leishmania*-specific ELISA antigens (Fig. 1B), although no significant correlation was found between any of these antigens and SECA.

The hierarchical clustering of serological responses in the FeL + group produced a dendrogram with two main branches (Fig. 1C): one grouping *Leishmania*-specific antigens (black branch) and another with the non-related antigen (red branch). The *Leishmania*-specific branch was further separated into two clusters: a blue cluster, grouping recombinant proteins K39, K28 and KDDR (with rKDDR separated from rK39 and rK28), and a green cluster, including SPLA and LicTXNPx.

To explore the structure and complementarity of serological responses across ELISA antigens, PCA was applied to the control dataset (Fig. 1D). The first two principal components accounted for 92.5% of the total variance, with PC1 alone explaining 84.6%, indicating that most variability in serological responses is captured along a single dominant axis. The PCA variable representation revealed two closely related antigen groups: recombinant kinesin antigens (rK39, rK28 and rKDDR), which clustered together and pointed in a similar direction, and SPLA and LicTXNPx, which also showed strong alignment with each other but in a distinct direction in the PCA space (Fig. 1D). This pattern reflects correlated but non-redundant serological responses among antigen classes.

To assess whether these serological patterns could be summarized into discrete groups, *k*-means clustering was applied to the PCA scores. The elbow method suggests that two or four clusters could group the ELISA data (Fig. 1E). The two-cluster option (*k* = 2) was deemed more informative, as it clearly separated FeL + and FeL − controls in two spatially distinct clusters (Fig. 1F). The four-cluster configuration (*k* = 4) (Additional file 3: Figure S1) distinctly subdivided positive and negative controls into four spatially discrete groups (two for the positives and two for the negatives).

### Cut-off approach to determine seropositivity to ELISA antigens and their associations to IFAT, DAT and PCR results

Clustering of the control ELISA dataset using two clusters (*k* = 2) exactly reproduced the predefined FeL + and FeL − groups (Fig. 1F**)**, supporting the use of quantitative thresholds to discriminate seropositive from seronegative samples. Seropositivity thresholds for each ELISA antigen were determined using a cut-off calculated as the mean OD value of FeL − samples + three standard deviations (Additional file 2: Table S2). The application of these cut-offs resulted in a mixture of seropositive and seronegative outcomes among individual FeL + controls (Additional file 2: Table S2). The rK39 and LicTXNPx were the most sensitive antigens, detecting all six FeL + samples as seropositive. The rKDDR failed to identify control E, while SPLA and rK28 presented lower Sn, with SPLA failing to detect FeL + controls C and E and rK28 misclassifying FeL + D and E. LicTXNPx was also found to be the least specific *Leishmania*-antigen, as it was the only antigen to classify a FeL − control as seropositive (Additional file 2: Table S2). IFAT and DAT correctly identified all FeL + and FeL − controls as seropositive and seronegative, respectively (Additional file 1: Table S1). No FeL − control sample was seropositive to more than one *Leishmania*-specific antigen, while all FeL + controls were seropositive to at least two antigens. Considering antigen performance and the hierarchical clustering of serological responses, the rK39 presented the best capacity to detect true-seropositive and true-seronegative controls (Fig. 1 C; Additional file 2: Table S2), standing out among the kinesin-related antigens. Together with LicTXNPx and SPLA—both distinct antigens to the kinesins—these three antigens provide a broad serological coverage.

**Fig. 2:**
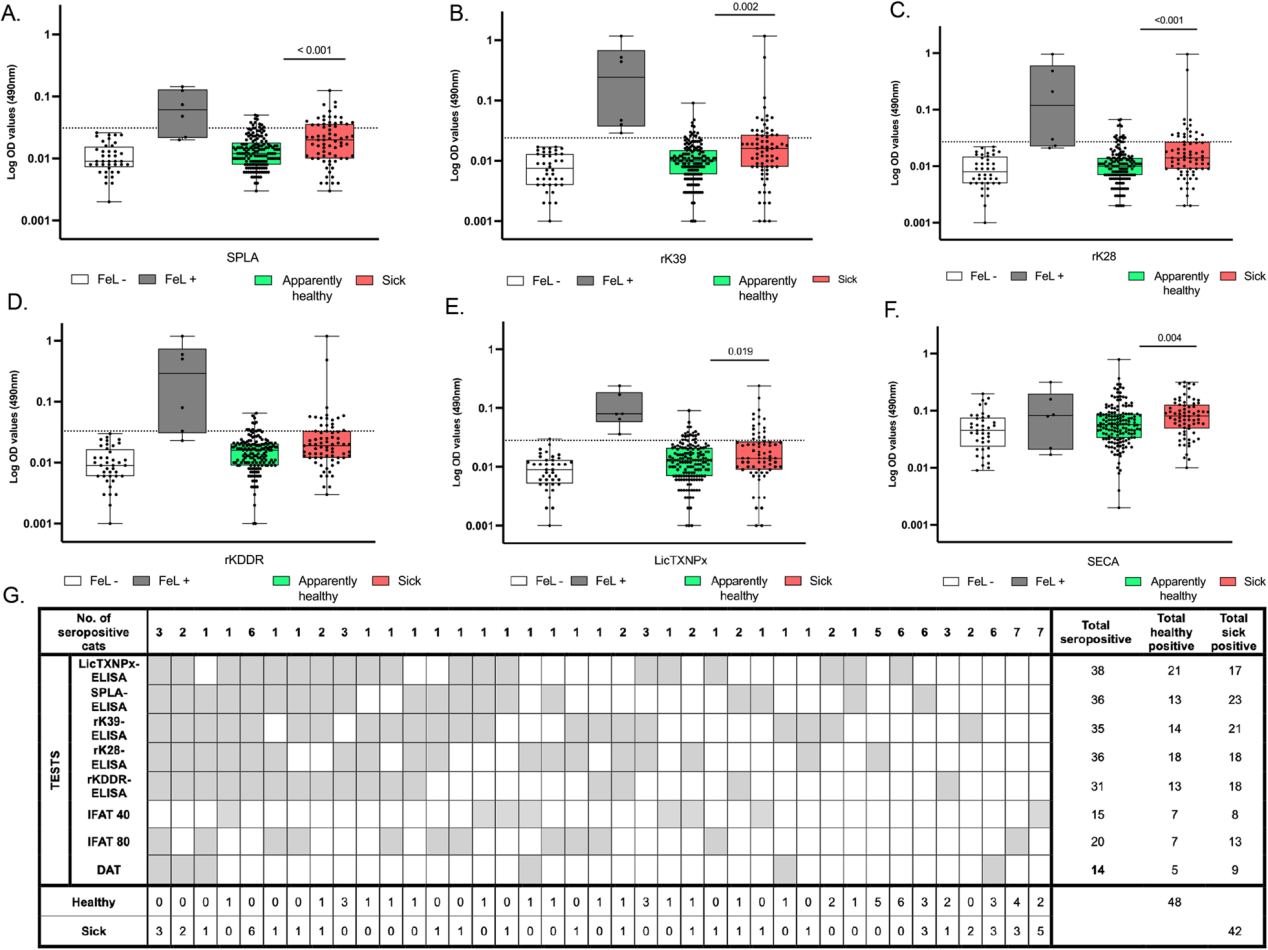
Distribution of seropositivity to the ELISA antigens, IFAT (40 and 80) and DAT in both the FeL + and FeL − controls and study group (*n* = 228), the latter divided into apparently healthy (*n* = 157) and sick (*n* = 71) cats. **A–F** Each box represents the interquartile range with the horizontal line indicating the median value. Whiskers extend to the minimum and maximum observed values. All individual observations are overlaid as black dots. Each dot represents the average of at least two independent assays for the same antigen for each sample. The Y-axis shows optical densities (OD) at 490 nm on a log 10 scale. The dotted line represents the cut-off value for each individual antigen. The studied antigens (A, SPLA; B, rK39; C, rK28; D, rKDDR; E, LicTXNPx; F, SECA) are depicted on the X-axis. Statistical comparisons between study groups were assessed by Mann-Whitney U-test. **G** Seropositivity to the ELISA tests SPLA, rK39, rK28, rKDDR, LicTXNPx and IFAT (cut-off 40 and 80) and DAT in the study cohort (*n* = 228) separated into healthy (*n* = 157) and sick (*n* = 71) cats. The grey shaded squares represent the positive results. The table on the right side represents the distribution of seropositivity to each test in healthy and sick cats. DAT, direct agglutination test; ELISA, enzyme-linked immunosorbent assay; FeL +, feline leishmaniosis-positive control; FeL −, feline leishmaniosis-negative control; IFAT, indirect fluorescent antibody test; LicTXNPx, *Leishmania infantum* recombinant cytosolic peroxiredoxin protein; rK28, *L. infantum* recombinant kinesin K28; rK39, *L. infantum* recombinant kinesin K39; rKDDR, *L. infantum* recombinant kinesin degenerated derived repeat; SECA, *Escherichia coli* soluble antigens; ns: not significant; SPLA, soluble promastigote *Leishmania* antigens

To evaluate the performance of the distinct ELISA propositions, a population of cats (*n* = 228) living in regions endemic for leishmaniosis was tested. Serological evaluation also included IFAT and DAT, and PCR was performed to detect *Leishmania* DNA in peripheral blood (*n* = 168). To assess the impact of clinical condition on seropositivity, cats in the study group were categorized as “apparently healthy” or “sick” (Fig. 2), as "sick" represents the main diagnostic target. For all antigens, significantly higher seroreactivity was observed in the group of sick cats, SPLA (*P* < 0.001), rK39 (*P* = 0.002), rK28 (*P* < 0.001), rKDDR (*P* = 0.003), LicTXNPx (*P* = 0.019) and SECA (*P* = 0.004), supporting enrichment of seropositive results in cats showing clinical signs of disease (Fig. 2).

For all serological approaches, seropositivity was higher for LicTXNPx (17%), followed by SPLA and rK28 (16%), rK39 (15%), rKDDR (14%), IFAT (cut-off: 80) (9%) and DAT (6%) (Fig. 2G). Considering seropositivity to any test, overall seroprevalence was as high as 40% (90/228) (Fig. 2G), while restricting it to *Leishmania*-specific ELISA antigens yielded 31% (70/228). PCR detected the lowest number of FeL (4%) (Additional file 4: Table S3). Seropositivity to any test was significantly overrepresented in sick cats (Fig. 2G, Additional file 4: Table S3). Seropositivity to three or more ELISA antigens was detected in 31 cats (13.6%), more frequently found among the sick (18/71, 25.4%) than in apparently healthy (13/157, 8.3%) cats (Fig. 2G; Additional file 4: Table S3). Notably, triple positivity to SPLA, rK39 and LicTXNPx—considered the minimal most predictive combination of antigens—was among the most enriched in sick animals (*χ*2 = 19.961, df = 1, *P* < 0.001). Seropositivity to all *Leishmania*-specific ELISA antigens was observed in 12/228 cats (5.3%), of which 11/12 (91.7%) were clinically sick. Among these, three animals were positive for IFAT (cut-off: 80) and DAT (cut-off: 100), two were only DAT-positive, and six were negative on IFAT and DAT. The only apparently healthy cat seropositive to all *Leishmania*-specific ELISA antigens was also IFAT 1:40 positive (Fig. 2G).

The disparity between individual tests and overall seropositivity prompted evaluation of agreement between tests using Cohen's kappa (*n* = 90; any sample seropositive to at least one test). Agreement among *Leishmania*-specific ELISAs ranged from fair [0.21–0.40] to moderate [0.41–0.60]. Agreement between DAT or IFAT and any *Leishmania*-specific ELISA antigen ranged from poor (*k* < 0) to slight (*k* < 0.2). Agreement between PCR and any *Leishmania*-specific ELISA or IFAT was slight (0 ≥ *k* < 0.20), and fair (0.21 ≥ *k* < 0.20) for DAT (Additional file 5: Table S4).

**Fig. 3:**
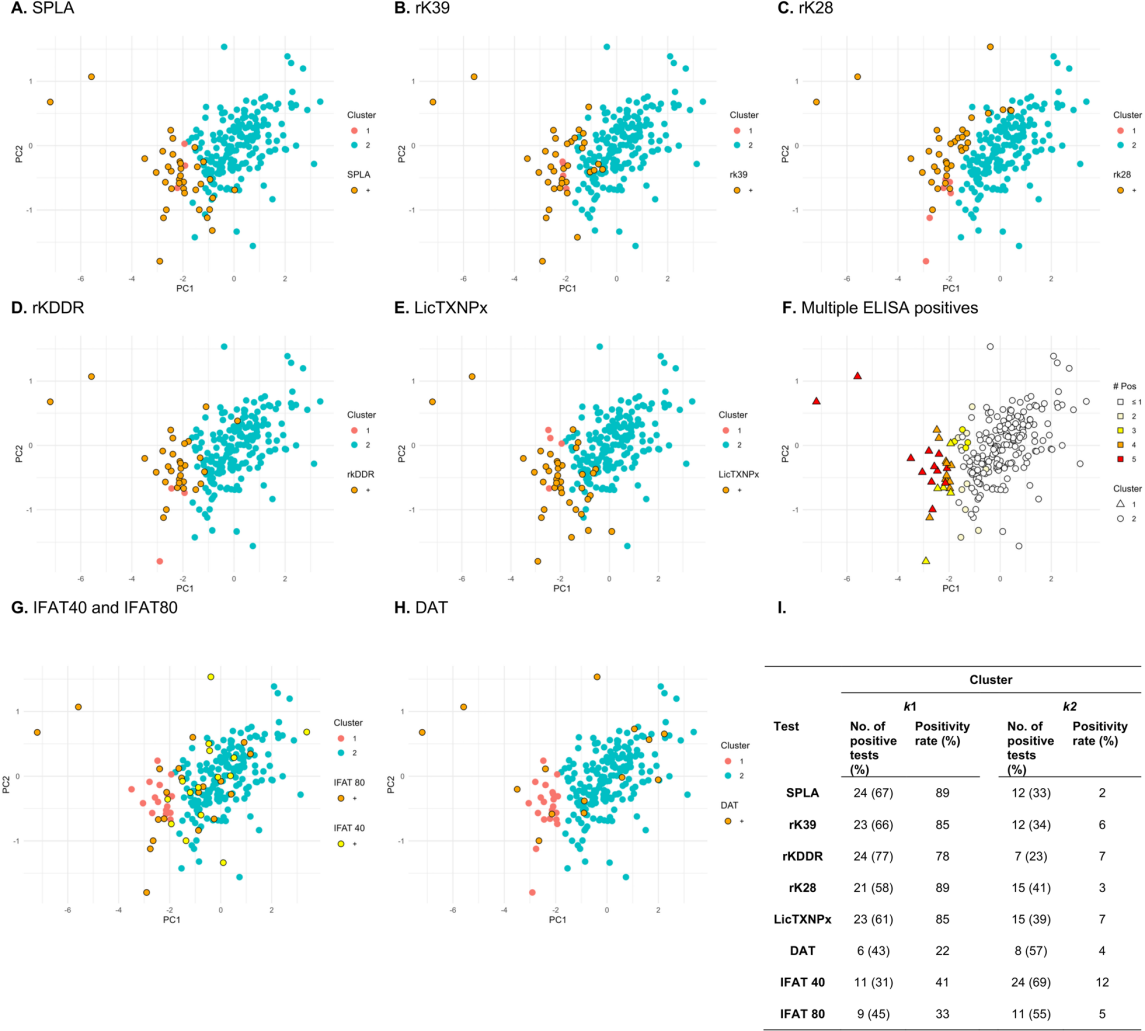
Two-dimensional representation of positive test results for *Leishmania*-specific ELISA antigens (SPLA, rK39, rK28, rKDDR, LicTXNPx), IFAT (cut-off 40 and 80) and DAT (cut-off 100) in the principal component analysis (PCA) space, distributed between *k*1 and *k*2 clusters. **A–E** Spatial distribution of *Leishmania*-specific ELISA antigen-positive results within clusters *k*1 and *k*2 of the PCA space. **F** Spatial distribution of samples seropositive to two or more ELISA antigens within clusters *k*1 and *k*2 of the PCA space. **G** Spatial distribution of IFAT 40 and IFA 80-positive results within clusters *k*1 and *k*2 of the PCA space. **H** Spatial distribution of DAT-positive results within clusters *k*1 and *k*2 of the PCA space. **I** Number and positivity (%) of seropositive results for each diagnostic test by clusters *k*1 and *k*2. DAT, direct agglutination test; ELISA, enzyme-linked immunosorbent assay; IFAT, indirect fluorescent antibody test; LicTXNPx*, Leishmania infantum* recombinant cytosolic peroxiredoxin protein; rK28, *Leishmania infantum* recombinant kinesin 28; rK39, *L. infantum* recombinant kinesin 39; rKDDR, *L. infantum* recombinant kinesin degenerated derived repeat; SPLA, soluble promastigote *Leishmania* antigens

Given the limited agreement, we examined how these animals were segregated under the *k*-clustering conditions defined for the control groups (Fig. 3A–E). Projection of single and multiple positive *Leishmania*-specific ELISA results into the PCA space (Fig. 3) revealed a clear concentration of seropositive cats in cluster *k*1 (Fig. 3I). All animals in *k*1 were seropositive for at least three distinct ELISA tests (Fig. 3F). In contrast, IFAT 1:40 and 1:80 or DAT presented a dispersed distribution across *k*1 and *k*2 clusters, being overall more common in *k*2 (Fig. 3G,H).

### Associations among ELISA, IFAT, DAT and PCR positivity with clinical findings, signalment data, haematological and serum biochemical parameters

Both traditional cut-offs and cluster analysis yielded a complex scenario, requiring multiple tests for more predictive serology. The clinical characterization of these animals, combined with distinct serological approaches, provides a unique opportunity to explore possible associations between positivity to single or multiple tests and biological alterations induced by *Leishmania* infection. To this end, clinical (Additional file 4: Table S3), haematological (Additional file 6: Table S5) and serum biochemistry abnormalities (Additional file 7: Table S6) were crossed with the serological data. Significant, correlations are summarized in Table 1.

**Table 1:**
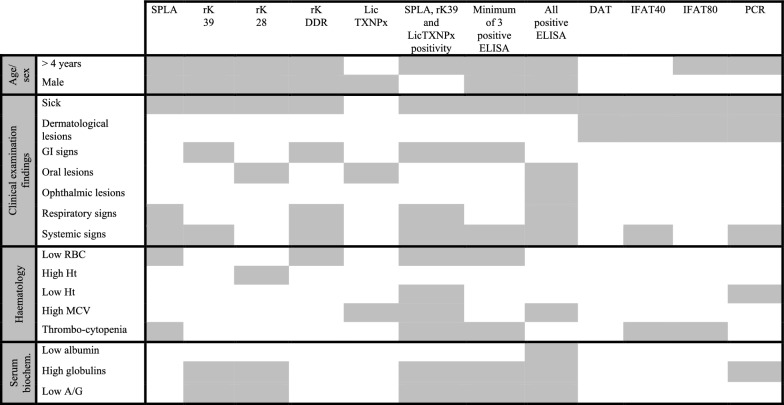
Summary of significant associations between positivity to ELISA antigens (SPLA, rK39, rK28, rKDDR and LicTXNPx), IFAT (cut-offs: 40 and 80), DAT and PCR and signalment (*n* = 212), clinical examination findings (*n* = 228), clinicopathological findings (haematology [*n* = 87]; serum biochemistry [*n* = 86]). Associations between variables were determined by chi-square or Fisher’s exact test (FET) analysis. Grey squares represent statistically significant associations (*P* < 0.05)

A/G, albumin/globulin ratio; DAT, direct agglutination test; ELISA, enzyme-linked immunosorbent assay; GI, gastrointestinal; Ht, haematocrit; IFAT, indirect fluorescent antibody test; LicTXNPx*, Leishmania infantum* recombinant cytosolic peroxiredoxin protein; MCV, mean cell volume; PCR, polymerase chain reaction; RBC, red blood cells; rK28, *L. infantum* recombinant kinesin 28; rk39, *L. infantum* recombinant kinesin 39; rKDDR, recombinant *L. infantum* kinesin degenerated derived repeat; SPLA, soluble promastigote *Leishmania* antigens

In this study group, FeL seropositivity to *Leishmania*-ELISA antigens, DAT and IFAT 1:40 was significantly associated with adult age (> 3 years old). Compared with other diagnostic tests, male cats were more frequently ELISA-seropositive (Table 1; Additional file 8: Table S7). Considering individual tests, statistically significant associations were observed between tests and the presence of clinical manifestations of disease, except for LicTXNPx (Table 1; Additional file 4: Table S3). These associations were particularly strong for systemic and unspecific signs of diseases (Table 1; Additional file 4: Table S3) and positivity to SPLA, rK39, rKDDR, IFAT 1:40 and PCR.

The presence of dermatological lesions was a distinguishing feature of IFAT-, DAT- and PCR-positive animals. Although no clear association was found between dermatological lesions and ELISA seropositivity (Table 1), six of seven (85.7%) IFAT 1:80-seropositive cats with dermatological lesions (Additional file 4: Table S3) were also positive for rK39, with five of them simultaneously positive for SPLA.

No distinct pattern was observed between FeL seropositivity and the presence of neurological or urinary signs of disease, compared with the general population (Additional file 4: Table S3). On the other hand, seropositivity to all *Leishmania-*specific ELISA antigens strongly predicted poorer clinical outcomes. Compared with the general FeL-seronegative population, this subgroup exhibited a stronger association with multiple clinical manifestations of disease, including systemic and respiratory signs as well as oral and ophthalmic lesions. Haematological abnormalities associated with this group included elevated mean red blood cell volume (MCV) (Table 1, Additional file 6: Table S5) and serum biochemistry findings included hypoalbuminaemia, hyperglobulinaemia and a consequent low A/G ratio (Additional file 7: Table S6). No distinguishing associations between abnormal erythrogram and/or serological findings were observed for IFAT-, DAT- or PCR-positive cats in relation to the general study populations (Additional file 6: Table S5; Additional file 7: Table S6). On the other hand, triple SPLA-rK39-LicTXNPx seropositive cats have the highest probability of being anaemic (low RBC, low Ht, high MCV) and thrombocytopenic (Table 1, Additional file 6: Table S5). A zoom-in over serum biochemistry abnormalities and FeL seropositivity revealed that only ELISA-seropositive samples were associated with hyperglobulinaemia, low A/G ratio and low albumin concentration (Additional file 7: Table S6). These associations were particularly stronger in cats presenting seropositivity to rK28 and rK39, triple seropositivity to SPLA-rK39-LicTXNPx or seropositivity to at least three *Leishmania*-specific antigens. Similarly, PCR positivity and hyperglobulinaemia were not independent features. The absence of statistically significant associations among a high concentration of albumin or total proteins, high serum levels of ALT, alkaline phosphatase, Crea or BUN and FeL seropositivity indicate that such clinical features do not distinguish FeL from other pathologies (Additional file 7: Table S6).

## Discussion

To improve understanding of the significance of serology in FeL studies, the diagnostic performance of a multi-antigen ELISA was compared with that of other serological assays (i.e. IFAT and DAT) and with molecular detection by PCR. Two complementary approaches were applied: (i) ELISA seropositivity determined by means of a cut-off computed from FeL– controls; (ii) an independent approach based on a cluster analysis. Both approaches provided strong support for the notion that *L. infantum*-infected cats produce specific antibodies against the parasite that are associated with clinical or clinicopathological alterations. Collectively, the data raise awareness about the need for cautious interpretation of single test results and support a multiparameter serological approach to improve FeL diagnosis.

The Sp of *Leishmania*-reactivity against the five *Leishmania* antigens is well supported by the use of SECA. This *Leishmania*-unrelated antigen has been previously used to highlight *Leishmania*-specific serological responses [42]. The average response to SECA in the FeL– cats was higher than that observed for other *Leishmania* antigens, supporting the absence of a *Leishmania*-related seroreactivity among *Leishmania*-naïve cats. Moreover, seroreactivity differences were observed when clinically sick cats were tested with *E. coli*-derived antigens. In fact, most of the sick feline population presented high seroreactivity to SECA—an expected finding, considering the likely scenario of sick animals being primarily or secondarily infected with bacteria.

Unlike CanL, where high antibody titers are often associated with disease severity [40, 42, 46, 47] and typically increase over the course of disease progression [48], our data suggest that humoral response in FeL is generally less pronounced and displays inconsistent patterns of seroreactivity between FeL clinical presentations. Compared to FeL– animals, FeL + controls presented significantly higher responses to *Leishmania* antigens. Nonetheless, signal-to-noise ratios were variable and generally low for each *Leishmania* ELISA. In fact, a distinctive finding of this study was the irregular dispersion of OD values among FeL + controls, ranging from near-baseline reactivities—almost indistinguishable from the naïve population (i.e. SPLA)—to markedly elevated OD values (i.e. rK39 and LicTXNPx). This variation was not present for SECA, the ubiquitous and non-*Leishmania*-related antigen. These inconsistencies contrast with findings from studies on CanL ELISA [40], where SPLA, rK39, rK28 and rKDDR have undoubtedly identified CanL-sick dogs (*n* = 121) with clinical and parasitological presentations similar to those described for the FeL + controls used in this study (i.e. clinical signs of leishmaniosis, positive DAT result, parasitological evidence of *Leishmania* infection).

Possible explanations for the variable antibody detection in cats could include possible shorter antibody half-life, reduced binding affinity, immune-exhaustion from chronic persistent infection, inherently weaker feline humoral response to *Leishmania* parasites infection and the immunosuppressive impact of FIV coinfection in FeL immune response. The influence of FIV and FeLV coinfections in FeL remains inconsistently reported across studies and geographical regions. This fact likely reflects geographical variations in epidemiological contexts [10, 29, 32, 37–39]. Nevertheless, in geographical areas endemic for both FIV and FeL, a positive association was found between both conditions, with FIV positive cats being at higher risk of FeL [13, 21, 30, 49]. Future studies are required to evaluate the dynamics of antibody response to *Leishmania* in the context of virus-induced immunosuppression.

Cluster analysis revealed two distinct immune response profiles: one primarily driven by recombinant kinesin antigens (i.e. rK39, rK28, rKDDR) and another associated with SPLA and LicTXNPx. Despite these differences, both recombinant kinesin antigens, SPLA and LicTXNPx, demonstrated high accuracy in detecting feline antibodies against *Leishmania*. Their diagnostic Sp is also supported by unrelated seroreactivity to SECA. This suggests that recombinant kinesin antigens identify a shared immune response, apparently distinct from that elicited by SPLA and LicTXNPx. Consequently, combining recombinant *Leishmania* kinesin antigens with SPLA and LicTXNPx should add diagnostic value and provide complementary information on the feline immune response to *Leishmania*.

The similarity in performance among *Leishmania* recombinant kinesin rK39, rK28 and rKDDR is expected, given their shared structural backbone with *L. infantum* kinesin proteins (e.g. TriTrypDB: Linj.14.1180). While rK39 antigen consists of a tandem repeat of the highly immunodominant 39-amino acid-long motif of *L. infantum* kinesin proteins, rk28 is a chimera of selected tandem repeat motifs from recombinant proteins K9, K26 and K39. rKDDR is almost entirely composed (~ 92%) of 8.5 blocks of the same 39-amino acid tandem repeats. It differs from rK39 by a unique 10-amino-acid non-repetitive segment along with a plasmid-derived sequence [50]. Although some studies report a higher Sn and Sp of rKDDR in humans and dogs [50], this was not observed in our FeL + cohort. Here, rK39 demonstrated superior diagnostic performance, correctly identifying all FeL + and FeL − controls, supporting a superior combination of Sn and Sp over the remaining antigens. This finding appears consistent with its ability to detect specific antibodies to *Leishmania* in CanL-sick dogs [40–42]. In contrast, SPLA antigen, derived from soluble cytosolic proteins of *Leishmania* promastigotes, failed to detect two of the six FeL + samples. This indicates SPLA's lower diagnostic Sn for FeL compared to CanL [40–42], an important finding since crude soluble promastigote *Leishmania* proteins have been widely used as ELISA antigens for sero-epidemiological surveys and FeL experimental infection studies [9, 13, 38, 39, 51]. LicTXNPx was sensitive but presented a lower Sp. For CanL, this antigen offered early recognition of antibodies to *Leishmania* in the context of experimental infection [52] and in animals without clinical signs [41]. This distinct recognition profile might also occur in cats. In natural *Leishmania* infections, as in the case of our FeL + controls, the time elapsed from infection is impossible to estimate. However, the lower LicTXNPx Sp in anti-*Leishmania* antibody detection in the FeL – cohort suggests cross-reactivity issues. Still, this antigen is a promising candidate for used in combination with other established ELISA antigens, especially the kinesins—highly recognised as specific markers for the diagnosis of diseases associated with CanL [41, 53].

Previous studies have reported variable agreement between SPLA-ELISA and IFAT results, ranging from almost perfect (*k* > 0.8) [39] to fair (0.2 > *k* < 0.41) [13], and between SPLA-ELISA and DAT (*k* = 0.80) [9]. In our study, only fair agreement was found between paired combinations of *Leishmania*-specific ELISA antigens, while other combinations of serological tests showed very poor concordance. This heterogeneity complicates interpretation of FeL serology, limiting the reliability of single-biomarker approaches. However, it may reflect variable immune responses during different stages of infection and disease progression, as previously described for CanL [40]. Over the course of CanL progression, differential expression of IgG isotypes can vary and may exhibit particular affinity for the antigens used in different serological tests [47]. In cats, only one IgG isotype (IgG1) with two alleles has been identified to date, with a putative IgG2 subclass lacking full characterization [54]. This limited immunoglobulin diversity may contribute to the inconsistent serological patterns observed in FeL.

The PCA analysis applied in this study provided insights into the performance of the antigens in FeL diagnosis by illustrating the relative contributions of the studied *Leishmania*-specific antigens to the dataset's variance. The positioning and proximity of all ELISA antigens on the same side of the PCA space indicate that they detect a common immune response pattern. Furthermore, PCA highlighted that multiple combinations of these antigens improved the interpretation of FeL-specific serological responses, either alone or in combination with IFAT and/or DAT. PCA showed a tendency for multiple ELISA-seropositive cats to group into a single cluster (*k*1). Furthermore, combinations of multi-antigen ELISA positivity with IFAT and/or DAT—also enriched in *k*1 cluster—constitute a pattern that collectively supports the link between highly reactive serological profiles and increased risk of FeL. These observations mirror findings described by Santarém et al. [41], where clustering facilitated the selection of antigens with complementary but non‑redundant diagnostic contributions to CanL, improving the interpretation and confirmation of *Leishmania*-specific serological responses.

Similarly to CanL, where disease-associated pathophysiological disorders correlate with anti-*Leishmania* antibody detection [15], we observed a comparable link between seropositivity and clinically relevant disease in cats. Significant associations were found between seropositivity to SPLA, rK39, rK28, rKDDR, IFAT or DAT and the presence of clinical signs of diseases. These were particularly evident in animals yielding multiple positive test results, supporting the hypothesis that diagnostic combinations create serological profiles consistent with distinct clinical features of FeL, as reported for CanL [15, 55]. Compared with other cats from the study population presenting variable health statuses, seropositivity to *Leishmania* was significantly associated with relevant clinical, haematological and serum biochemistry abnormalities related to FeL. In fact, clinicopathological alterations associated with disease progression and poor prognosis, namely anaemia and thrombocytopenia, hypergammaglobulinaemia and low A/G ratio (reflecting low albumin and high globulin serum concentrations), were strongly associated with seropositivity to multiple *Leishmania*-specific ELISA antigens. Our findings are in line with previous reports [20], highlighting distinguishing features between seropositive cats and the wider study population. In fact, hypergammaglobulinaemia was considered a relevant clinicopathological abnormality associated with FeL (reviewed by Garcia-Torres et al. [20] and analysed by Carbonara et al. [13]), likely representing a laboratory marker predictive of disease among seropositive cats. Notably, such patterns were not observed in IFAT- or DAT-positive cats.

As in CanL, FeL presents as a syndromic disease typically involving multiple organ systems and lacking pathognomonic signs. A recent review found that > 58% of reported FeL cases presented with multiple clinical signs, most frequently dermatological lesions, followed by systemic, ocular, mucocutaneous and respiratory manifestations [20]. Here, FeL seropositivity was significantly associated with adult (> 3 years) and male cats, consistent with previous reports from northern Portugal [9], Spain [30] and Italy [49]. Cutaneous lesions were a particularly distinguishing feature in IFAT- and DAT-positive cats compared with the overall study population. Although no statistically significant associations were found between seropositivity to *Leishmania*-specific ELISA antigens and dermatological signs, most IFAT- and DAT-positive cats with dermatological signs were also rk39-positive. Conversely, seropositivity to all *Leishmania*-specific ELISA antigens was associated with a more diverse clinical profile, including oral and/or ophthalmic lesions, as well as respiratory and/or systemic signs of disease.

The grounds for supporting rK39, SPLA and LicTXNPx in serological approaches to CanL diagnosis and serological surveys are well established [40–42]. Our results highlight rK39's high accuracy in detecting clinical cases of FeL. Moreover, the results suggest that seropositivity to rK39 in complementary combination with SPLA and LicTXNPx seropositivity increases detection of samples with strong association to relevant clinical markers of disease (including anaemia, thrombocytopenia, hyperglobulinaemia and a low A/G ratio). Therefore, a combination of seropositivity to rK39, SPLA and LicTXNPx increases the positive predictive value of a seropositive result. The availability of rK39 and SPLA for CanL diagnosis, coupled with the feasibility of large‑scale recombinant production of LicTXNPx, strengthens the three biomarkers' potential for future FeL diagnostic strategies.

The comparatively lower Sn of blood-based PCR compared with serological antibody detection has been widely reported in FeL epidemiological studies [10, 13, 27, 30, 49]. Biologically, this discrepancy may be justified by parasite tissue-tropism and variable burden of infection over the course of disease [56]. While serum antibodies may persist for months following exposure, parasite detection relies on the chances of capturing parasitized cells [57] in the sampled tissues at the time of sampling [58]. From a technical standpoint, the analytical Sn of PCR depends not only on the presence of parasite DNA in the selected matrix but also on sample quality and quantity, as well as the efficiency of DNA extraction and amplification protocols [59]. In the context of this study, PCR positivity corroborated the susceptibility of this study population to *Leishmania* infection and further supported an association between PCR positivity and clinical disease. Specifically, PCR positivity was significantly associated with manifestations of severe systemic disease, including anaemia (low Ht) and hyperglobulinaemia. Nonetheless, the agreement between positivity to PCR and a serological test was low (< 0.2), given the low Sn of blood PCR.

Key limitations to this study included the suboptimal PCR Sn associated with blood (which might have limited the detection of infected animals) and the reduced number of FeL + controls included (which constrained the analysis of serological profiles related to *L. infantum* infection and disease). Concerning PCR, it is known that amplification of *Leishmania* DNA directly from mononuclear phagocytic system tissues (bone marrow, lymph node or spleen) is indeed more sensitive. These limitations are justified by the challenges of obtaining invasive tissue specimens under field conditions, the rarity of FeL diagnosis in clinical practice, and the limited probability of investing in both direct and indirect parasitological diagnoses to confirm overt FeL cases. Despite the reduced number of FeL + controls, these provided evidence over a diverse set of serological profiles associated with FeL and supported robust PCA analyses. Nevertheless, they were insufficient to reliably estimate positivity thresholds using receiver-operating characteristic (ROC) curve analysis. To further our understanding of antibody dynamics and their association with different FeL serological profiles, it is necessary to conduct longitudinal follow-up of seropositive cats, including assessment for coinfections and evaluation of progression toward FeL-associated disease or spontaneous resolution. However, such follow-up is impractical in field-based epidemiological studies.

## Conclusions

The elusive nature of FeL, along with its varied clinical manifestations, the lack of sufficiently pathognomonic clinical signs, and its frequent association with coinfections, poses diagnostic and therapeutic challenges. In this study, we demonstrated that *Leishmania*-infected cats can produce a specific antibody response, detectable by a variety of serological tests. This study also demonstrates that FeL serology benefits from multi-antigen strategies anchored in rK39, SPLA and LicTXNPx, with clustering corroborating complementary diagnostic information. Seropositivity—especially across multiple ELISA antigens—aligns with clinically meaningful abnormalities (haematological and biochemical) and richer clinical phenotypes, while single tests (such as blood PCR) show limited standalone utility. Future studies employing cut-offs derived from larger and geographically dispersed FeL-positive and -negative cohorts should enable more robust results and a more stringent assessment of true Sn and Sp. Moreover, isotype-aware assay design and prospective longitudinal cohorts will also be instrumental to optimizing FeL diagnosis and clarifying antibody kinetics, particularly in the setting of FIV/FeLV coinfections.

## Supplementary Information

Additional file 1.

Additional file 2.

Additional file 3.

Additional file 4.

Additional file 5.

Additional file 6.

Additional file 7.

Additional file 8.

## Data Availability

The authors confirm that the data supporting the findings of this study are available within the article and its supplementary materials.

## Abbreviations

A/G: Albumin/globulin ratio
ALT: Alanine aminotransferase
BUN: Blood urea nitrogen
CanL: Canine leishmaniosis
Crea: Creatinin
DAT: Direct agglutination test
ELISA: Enzyme-linked immunosorbent assay
FeL: Feline leishmaniosis
FeLV: Feline leukaemia virus
FET: Fisher’s exact test
FIV: Feline immunodeficiency virus
Ht: Haematocrit
IgG: Immunoglobulin
IFAT: Indirect fluorescent antibody test
LicTXNPx: *Leishmania* Cytosolic peroxiredoxins
MCV: Mean cell volume
PCA: Principal component analysis
PCR: Polymerase chain reaction
RBC: Red blood cells
rK28: Recombinant *Leishmania* kinesin 28
rK39: Recombinant *Leishmania* kinesin 39
rKDDR: Recombinant *Leishmania* kinesin degenerated derived repeats
Sn: Sensitivity
SECA: Soluble *Escherichia coli* antigens
Sp: Specificity
SPLA: Soluble promastigote *Leishmania* antigens
