# Supplementary material for: Diagnostic potential of a multi-antigen ELISA for feline leishmaniosis
Source: Parasit Vectors. 2026 Mar 16;19:157. doi: 10.1186/s13071-026-07320-5 (PMC13077857; doi:10.1186/s13071-026-07320-5)
Supplement: Supplementary file 1 — Additional file 1. [file 13071_2026_7320_MOESM1_ESM.docx]

**Additional file 1: Table S1** Clinical and diagnostic laboratory characterization of feline leishmaniosis (FeL) positive controls.

| Control | Clinical characterization of FeL | FIV | FeLV | DAT | IFAT | Parasitological detection of *Leishmania* spp. |
| --- | --- | --- | --- | --- | --- | --- |
| A | Dermatological lesions, severe anaemia, cachexia | + | + | + | + | Positive PCR and culture |
| B | Dermatological and ocular lesions; favourable response to alopurinol | − | − | + | + | Positive PCR and culture |
| C | Ocular lesions; favourable response to antimonials and alopurinol | − | − | + | + | Positive PCR and cytology |
| D | Ocular lesions; favourable response to antimonials and alopurinol | + | − | + | + | Negative PCR, positive cytology |
| E | Ocular lesions; favourable response to alopurinol | nd | nd | + | + | Negative PCR |
| F | Dermatological lesions; favourable response to alopurinol | − | − | + | + | nd |

DAT, direct agglutination test (cut-off: 100); FeLV, feline leukaemia virus; FIV, feline immunodeficiency virus; IFAT, indirect fluorescent antibody test (cut-off: 80); PCR, polymerase chain reaction; +, positive; −, negative; nd, not determined.
