# Supplementary material for: Diagnostic potential of a multi-antigen ELISA for feline leishmaniosis
Source: Parasit Vectors. 2026 Mar 16;19:157. doi: 10.1186/s13071-026-07320-5 (PMC13077857; doi:10.1186/s13071-026-07320-5)
Supplement: Supplementary file 2 — Additional file 2. [file 13071_2026_7320_MOESM2_ESM.docx]

**Additional file 2: Table S2** Cut-off and seropositivity data to the different ELISA antigens (*Leishmania*-specific – SPLA, rK39, rK28, rKDDR, LicTXNPx) applied to study feline leishmaniosis positive (FeL +, n $=$6) and negative (FeL –, n$=$40) controls.

|  |  | FeL + | | | | | | FeL − |
| --- | --- | --- | --- | --- | --- | --- | --- | --- |
| Antigen | Cut-off value | A | B | C | D | E | F |  |
| SPLA | 0.031 | 0.048 | 0.125 | 0.022 | 0.144 | 0.020 | 0.074 | 0/40 |
|  |  | + | + | – | + | – | + |  |
| rK39 | 0.024 | 0.040 | 1.172 | 0.441 | 0.047 | 0.029 | 0.521 | 0/40 |
|  |  | + | + | + | + | + | + |  |
| rK28 | 0.027 | 0.030 | 0.957 | 0.209 | 0.021 | 0.023 | 0.485 | 0/40 |
|  |  | + | + | + | – | – | + |  |
| rKDDR | 0.033 | 0.033 | 1.187 | 0.594 | 0.060 | 0.031 | 0.504 | 0/40 |
|  |  | + | + | + | + | – | + |  |
| LicTXNPx | 0.028 | 0.079 | 0.240 | 0.036 | 0.080 | 0.169 | 0.065 | 1/40 |
|  |  | + | + | + | + | + | + |  |

ELISA, enzyme-linked immunosorbent assay; LicTXNPx*, Leishmania infantum* recombinant cytosolic peroxiredoxin protein; rK28, *L. infantum* recombinant kinesin 28; rK39, *L. infantum* recombinant kinesin 39; rKDDR, *L. infantum* recombinant kinesin degenerated derived repeat; SPLA, soluble promastigote *Leishmania* antigens; (+) positive; (–) negative; x̄, average. The positive control was identified by A, B, C, D and E.
