# Supplementary material for: Diagnostic potential of a multi-antigen ELISA for feline leishmaniosis
Source: Parasit Vectors. 2026 Mar 16;19:157. doi: 10.1186/s13071-026-07320-5 (PMC13077857; doi:10.1186/s13071-026-07320-5)
Supplement: Supplementary file 3 — Additional file 3. [file 13071_2026_7320_MOESM3_ESM.docx]

**Additional file 3: Figure S1** Partition of the control data set into four *k*-clusters, as estimated by the Elbow Method.


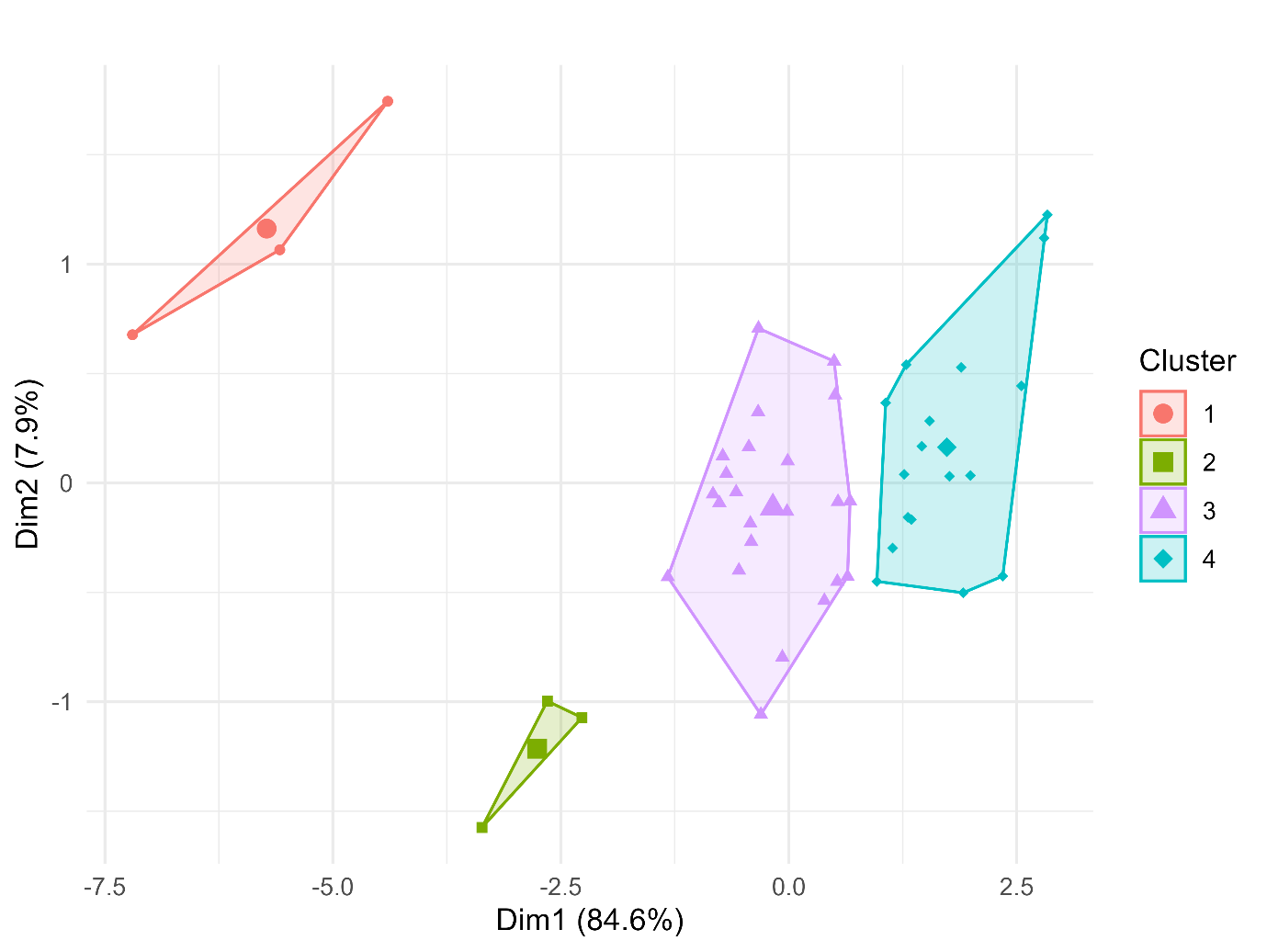


Dim, dimension.
