# Supplementary material for: Diagnostic potential of a multi-antigen ELISA for feline leishmaniosis
Source: Parasit Vectors. 2026 Mar 16;19:157. doi: 10.1186/s13071-026-07320-5 (PMC13077857; doi:10.1186/s13071-026-07320-5)
Supplement: Supplementary file 4 — Additional file 4. [file 13071_2026_7320_MOESM4_ESM.docx]

**Additional file 4: Table S3** Positivity to single or combination of tests in the study population and respective associations with clinical examination findings, given by Chi-square (χ^2^) or Fisher´s exact test (FET). Diagnostic tests included ELISA antigens (SPLA, rK39, rK28, rKDDR and LicTXNPx), DAT, IFAT40, IFAT80 and PCR.

| Diagnostic tests | | SPLA | rK39 | rK28 | rKDDR | LicTXNPx | SPLA, rK39 and LicTXNPx positivity | Minimum of 3 positive ELISA | All ELISA positive | DAT | IFAT40 | IFAT80 | PCR |
| --- | --- | --- | --- | --- | --- | --- | --- | --- | --- | --- | --- | --- | --- |
| No. of total seropositive/  total tested cats (%) | | 36/228 | 35/228 | 36/228 | 31/228 | 38/228 | 17/228 | 31/228 | 12/228 | 14/228 | 35/228 | 20/228 | 6/168 |
|  |  | (15.8) | (15.4) | (15.8) | (13.6) | (17.0) | (7.5) | (13.6) | (5.3) | (6.1) | (15.4) | (8.8) | (3.6) |
| No. of total sick and *Leishmania* seropositive cats (%) | | 23 (63.9) | 21 (60.0) | 18 (50.0) | 18 (58.1) | 17 (44.7) | 14 (82.4) | 18 (58.1) | 11 (91.7) | 9 (64.3) | 21 (60.0) | 13 (65.0) | 5 (83.3) |
|  |  | χ^2^ =19.606 | χ^2^ =14.509 | χ^2^ =6.085 | χ^2^ =10.719 | χ^2^ =3.207 | χ^2^ =19.961 | χ^2^ =10.719 | χ^2^ =18.763 | χ^2^ =6.084 | χ^2^ =14.509 | χ^2^ =10.054 |  |
|  |  | *P*<0.001* | *P*<0.001* | *P =* 0.014* | *P =* 0.001* | *P =* 0.073 | *P*<0.001* | *P =* 0.001* | *P<*0.001* | *P =* 0.014* | *P<*0.001* | *P =* 0.001* | *P^#^* = 0.021* |
| Clinical examination findings | Skin lesions (n) | 7 | 7 | 5 | 4 | 4 | 4 | 5 | 3 | 5 | 9 | 7 | 3 |
|  |  | *P^#^* = 0.085 | *P^#^* = 0.077 | *P^#^* = 0.562 | *P^#^* = 0.756 | *P^#^* = 1.0 | *P^#^* = 0.100 | *P^#^* = 0.351 | *P^#^* = 0.132 | *P^#^* = 0.011* | *P^#^* = 0.006* | *P^#^* = 0.002* | *P^#^* = 0.022* |
|  | Gastro-intestinal (n) | 2 | 3 | 1 | 2 | 2 | 2 | 2 | 1 | 0 | 0 | 0 | 0 |
|  |  | *P^#^* = 0.066 | *P^#^* = 0.003* | *P^#^* = 0.404 | *P^#^* = 0.049* | *P^#^* = 0.073 | *P^#^* = 0.015* | *P^#^* =0.049* | *P^#^* = 0.150 | *P^#^* = 1.0 | *P^#^* = 1.0 | *P^#^* = 1.0 | *P^#^* = 1.0 |
|  | Neurological (n) | 1 | 1 | 1 | 1 | 1 | 1 | 1 | 1 | 0 | 0 | 0 | 0 |
|  |  | *P^#^* = 0.291 | *P^#^* = 0.284 | *P^#^* = 0.291 | *P^#^* = 0.254 | *P^#^* = 0.306 | *P^#^* = 0.144 | *P^#^* = 0.254 | *P^#^* = 0.103 | *P^#^* = 1.0 | *P^#^* = 1.0 | *P^#^* = 1.0 | *P^#^* = 1.0 |
|  | Oral (n) | 4 | 3 | 5 | 4 | 5 | 3 | 4 | 3 | 0 | 4 | 2 | 1/4 |
|  |  | *P^#^* = 0.130 | *P^#^* = 0.427 | *P^#^* = 0.037* | *P^#^* = 0.083 | *P^#^* = 0.046* | *P^#^* = 0.062 | *P^#^* = 0.083 | *P^#^* = 0.024* | *P^#^* = 1.0 | *P^#^* = 0.120 | *P^#^* = 0.318 | *P^#^* = 0.115 |
|  | Ophthalmic (n) | 2 | 2 | 2 | 2 | 2 | 2 | 2 | 2 | 1 | 1 | 1 | 0/4 |
|  |  | *P^#^* = 0.241 | *P^#^* = 0.231 | *P^#^* = 0.241 | *P^#^* = 0.189 | *P^#^* = 0.262 | *P^#^* = 0.066 | *P^#^* = 0.189 | *P^#^* = 0.034* | *P^#^* = 0.319 | *P^#^* = 1.0 | *P^#^* = 0.427 | *P^#^* = 1.0 |

**Additional file 4: Table S3** (continued).

| Diagnostic tests | | SPLA | rK39 | rK28 | rKDDR | LicTXNPx | SPLA, rK39, LicTXNPx | Minimun of. 3 positive ELISA | All ELISA positive | DAT | IFAT40 | IFAT80 | PCR |
| --- | --- | --- | --- | --- | --- | --- | --- | --- | --- | --- | --- | --- | --- |
| No. of total seropositive/  total tested cats (%) | | 36/228 | 35/228 | 36/228 | 31/228 | 38/228 | 17/228 | 31/228 | 12/228 | 14/228 | 35/228 | 20/228 | 6/168 |
|  |  | (15.8) | (15.4) | (15.8) | (13.6) | (17.0) | (7.5) | (13.6) | (5.3) | (6.1) | (15.4) | (8.8) | (3.6) |
| Clinical examination findings | Respiratory (n) | 5 | 3 | 3 | 4 | 4 | 3 | 3 | 3 | 1 | 3 | 0 | 1 |
|  |  | *P^#^* = 0.017* | *P^#^* = 0.381 | *P^#^* = 0.387 | *P^#^* = 0.047* | *P^#^* = 0.090 | *P^#^* = 0.039* | *P^#^* = 0.176 | *P^#^* = 0.015* | *P^#^* = 0.510 | *P^#^* = 0.381 | *P^#^* = 0.605 | *P^#^* = 0.254 |
|  | Systemic (n) | 16 | 14 | 10 | 11 | 11 | 10 | 12 | 7 | 5 | 12 | 7 | 4 |
|  |  | χ^2^= 13.156 | χ^2^= 8.148 | χ^2^ = 0.871 | χ^2^ = 3.853 | χ^2^= 1.372 |  | χ^2^ = 5.956 |  |  | χ^2^= 3.787 |  |  |
|  |  | *P <* 0.001* | *P* = 0.004* | *P* = 0.264 | *P* = 0.050* | *P* = 0.179 | *P^#^ <*0.001* | *P =* 0.012* | *P^#^* = 0.004* | *P^#^* = 0.172 | *P =* 0.052 | *P^#^* = 0.142 | *P^#^* = 0.023* |
|  | Urinary (n) | 1 | 1 | 1 | 1 | 1 | 1 | 1 | 1 | 0 | 0 | 0 (0) | 0 (0) |
|  |  | *P^#^* = 0.500 | *P^#^* = 0.489 | *P^#^* = 0.500 | *P^#^* = 0.445 | *P^#^* = 0.520 | *P^#^* = 0.268 | *P^#^* = 0.444 | *P^#^* = 0.196 | *P^#^* = 1.0 | *P^#^* = 1.0 | *P^#^* = 1.0 | *P^#^* = 1.0 |
|  | Poor body condition^a^ (n) | 8 | 7 | 7 | 7 | 6 | 5 | 6 | 5 | 4 | 6 | 5 (25) | 4 (67) |
|  |  | χ^2^= 0.419 | χ^2^= 0.063 | χ^2^= 0.027 | χ^2^= 0.377 | χ^2^= 0.0 | *P^#^* = 0.180 | χ^2^= 0.010 |  |  | χ^2^= 0.0 |  |  |
|  |  | *P* = 0.517 | *P* = 0.802 | *P* = 0.869 | *P* = 0.539 | *P* = 1.0 |  | *P* = 0.919 | *P^#^* = 0.036* | *P^#^* = 0.268 | *P* = 1.0 | *P^#^* = 0.351 | *P^#^* = 0.007* |

DAT, direct agglutination test; ELISA, enzyme-linked immunosorbent assay; IFAT, indirect fluorescent antibody test; LicTXNPx*, L. infantum* recombinant cytosolic peroxiredoxin protein; PCR, polymerase chain reaction; rK28, *L. infantum* recombinant kinesin 28; rK39, *L. infantum* recombinant kinesin 39; rKDDR, *L. infantum* recombinant kinesin degenerated derived repeat; SPLA, soluble promastigote *Leishmania* antigens.

χ^2^ and FET (^#^) computed for binomial distribution; *df* = 1 for all χ^2^ measurements.

*Statistically significant difference.

^a^ Poor body condition defined by cachexia or underweight or previous history of weight loss, when documented.
