# Supplementary material for: Diagnostic potential of a multi-antigen ELISA for feline leishmaniosis
Source: Parasit Vectors. 2026 Mar 16;19:157. doi: 10.1186/s13071-026-07320-5 (PMC13077857; doi:10.1186/s13071-026-07320-5)
Supplement: Supplementary file 5 — Additional file 5. [file 13071_2026_7320_MOESM5_ESM.docx]

**Additional file 5: Table S4** Agreement beyond chance by Cohen´s kappa coefficient (*k*) in cats (n = 90) presenting at least one seropositive test (SPLA-ELISA, rK39-ELISA, rK28-ELISA, rKDDR-ELISA, LicTXPNPx-ELISA, IFAT [40 and 80 cut-offs] and DAT.

| Total positive results (n) | rK39 | rK28 | rKDDR | LicTXNPx | DAT | IFAT | | PCR |
| --- | --- | --- | --- | --- | --- | --- | --- | --- |
|  |  |  |  |  |  | 40 | 80 |  |
| SPLA (36) | *k* = 0.279  *P* = 0.008* | *k* = 0.279  *P* = 0.014* | *k* = 0.502  *P* < 0.001* | *k* = 0.358  *P* < 0.001* | *k* = 0.021  *P* = 0.812 | *k* = -0.047  *P* = 0.659 | *k* = 0.050  *P* = 0.605 | *k* = 0.099  *P* = 0.060 |
| rK39 (35) |  | *k* = 0.419  *P* < 0.001* | *k* = 0.475  *P* < 0.001* | *k* = 0.425  *P* < 0.001* | *k* = 0.082  *P* = 0.353 | *k* = -0.075  *P* = 0.475 | *k* = 0.113  *P* = 0.248 | *k* = 0.099  *P* = 0.060 |
| rK28 (36) |  |  | *k* = 0.408  *P* < 0.001* | *k* = 0.220  *P* = 0.037* | *k* = 0.072  *P* = 0.406 | *k* = -0.093  *P* = 0.377 | *k* = 0.0  *P* = 1.000 | *k* = 0.127  *P* = 0.032* |
| rKDDR (31) |  |  |  | *k* = 0.369  *P* < 0.001* | *k* = 0.067  *P* = 0.471 | *k* = -0.146  *P* = 0.164 | *k* = 0.060  *P* = 0.553 | *k* = 0.136  *P* = 0.026* |
| LicTXNPx (38) |  |  |  |  | *k* = -0.045  *P* = 0.592 | *k* = -0.128  *P* = 0.224 | *k* = -0.022  *P* = 0.820 | *k* = 0.093  *P* = 0.068 |
| DAT (14) |  |  |  |  |  | *k* = -0.023  *P* = 0.791 | *k* = 0.064  *P* = 0.534 | *k* = 0.227  *P* = 0.003* |
| IFAT 40 (28) |  |  |  |  |  |  |  | *k =* 0.083  *P* = 0.086 |
| IFAT 80 (20) |  |  |  |  |  |  |  | *k =* 0.155  *P* = 0.017* |

Association between seropositivity to at least one serological test and PCR result is presented for 68 samples. The level of agreement is represented by the colour of the cells: green for poor to slight (0 > *k* < 0.2), yellow for fair (0.21 ≥ *k* < 0.40) and orange for moderate (0.41 ≥ *k* < 0.60).

DAT, direct agglutination test; ELISA, enzyme-linked immunosorbent assay; IFAT, indirect fluorescent antibody test; LicTXNPx*, Leishmania infantum* recombinant cytosolic peroxiredoxin protein; PCR, polymerase chain reaction; rK28, *L. infantum* recombinant kinesin 28; rK39, *L. infantum* recombinant kinesin 39; rKDDR, *L. infantum* recombinant kinesin degenerated derived repeat; SECA, *Escherichia coli* soluble antigens; SPLA, soluble promastigote *Leishmania* antigens.

*Statistically significant difference.
