# Supplementary material for: Diagnostic potential of a multi-antigen ELISA for feline leishmaniosis
Source: Parasit Vectors. 2026 Mar 16;19:157. doi: 10.1186/s13071-026-07320-5 (PMC13077857; doi:10.1186/s13071-026-07320-5)
Supplement: Supplementary file 6 — Additional file 6. [file 13071_2026_7320_MOESM6_ESM.docx]

**Additional file 6: Table S5** Associations between abnormal feline haematological parameters and a positive result obtained for ELISA antigens (SPLA, rK39, rK28, rKDDR and LicTXNPx), DAT, IFAT (40 and 80) (n = 87) or PCR (n = 77) in the study group, given by Chi-square (χ2) or Fisher’s exact test (FET).

| Hematological parameters ^a^ | Tests | | | | | | | | | | | |
| --- | --- | --- | --- | --- | --- | --- | --- | --- | --- | --- | --- | --- |
|  | SPLA | rK39 | rK28 | rKDDR | LicTXNPx | SPLA, rK39 and LicTXNPx positivity | Minimum of 3 positive ELISA | All ELISA positive | DAT | IFAT40 | IFAT80 | PCR |
|  | No. of cats affected by each haematological abnormality/Total no. of positive tests | | | | | | | | | | | |
| Eritrogram |  |  |  |  |  |  |  |  |  |  |  |  |
| High RBC | 1/12 | 2/17 | 2/14 | 2/13 | 2/16 | 0/6 | 1/12 | 0/3 | 1/7 | 4/20 | 3/13 | 0/6 |
| 13/87 | *P^#^* = 1.0 | *P^#^ =* 1.0 | *P^#^* = 1.0 | *P^#^* = 1.0 | *P^#^* = 1.0 | *P^#^* = 0.590 | *P^#^* = 1.0 | *P^#^* = 1.0 | *P^#^* = 1.0 | *P^#^* = 0.462 | *P^#^* = 0.380 | *P^#^* = 1.0 |
| Low RBC | 3/12 (25) | 3/17(18) | 1/14 (7) | 3/13 (23) | 3/16 (19) | 3/6 (50) | 3/12 (25) | 1/3 (33) | 1/7 (14) | 2/20 (10) | 2/13 (15) | 2/6 (33) |
| 6/87 | *P^#^ =* 0.033* | *P^#^ =* 0.089 | *P^#^* = 1.0 | *P^#^* = 0.041* | *P^#^* = 0.073 | *P^#^* = 0.003* | *P^#^* = 0.033* | *P^#^* = 0.195 | *P^#^* = 0.405 | *P^#^* = 0.620 | *P^#^* = 0.223 | *P^#^* = 0.068 |
| High Ht | 1/12 | 3/17 | 0/14 | 1/13 | 3/16 | 1/6 | 2/12 | 0/3 | 1/7 | 5/20 | 3/13 | 0/6 |
| 19/87 | *P^#^* = 0.446 | *P^#^* = 0.754 | *P^#^* = 0.035* | *P^#^* = 0.282 | *P^#^* = 1.0 | *P^#^* = 1.0 | *P^#^* = 1.0 | *P^#^* = 1.0 | *P^#^* = 1.0 | *P^#^* = 0.754 | *P^#^* = 1.0 | *P^#^* = 0.582 |
| Low Ht | 2/12 | 2/17 | 1/14 | 2/13 | 2/16 | 2/6 | 2/12 | 1/3 | 1/7 | 1/20 | 1/13 | 2/6 |
| 5/87 | *P^#^* = 0.141 | *P^#^* = 0.251 | *P^#^* = 1.0 | *P^#^* = 0.159 | *P^#^* = 0.227 | *P^#^* = 0.037* | *P^#^* = 0.141 | *P^#^* = 0.164 | *P^#^* = 0.349 | *P^#^* = 1.0 | *P^#^* = 0.569 | *P^#^* = 0.047* |
| High Hg | 1/11 | 2/14 | 1/13 | 1/11 | 2/13 | 1/5 | 2/10 | 0/3 | 1/4 | 3/17 | 2/10 | 1/3 |
| 9/84 | *P^#^* = 1.0 | *P^#^* = 0.644 | *P^#^* = 1.0 | *P^#^* = 1.0 | *P^#^* = 0.624 | *P^#^* = 0.441 | *P^#^* = 0.296 | *P^#^* = 1.0 | *P^#^* = 0.370 | *P^#^* = 0.381 | *P^#^* = 0.296 | *P^#^* = 0.230 |
| Low Hg | 1/11 | 1/14 | 1/13 | 1/11 | 1/13 | 1/5 | 1/10 | 1/3 | 0/4 | 0/17 | 0/10 | 1/3 |
| 4/84 | *P^#^* = 0.436 | *P^#^* = 0.530 | *P^#^* = 0.501 | *P^#^* = 0.436 | *P^#^* = 0.496 | *P^#^* = 0.221 | *P^#^* = 0.408 | *P^#^* = 0.138 | *P^#^* = 1.0 | *P^#^* = 0.577 | *P^#^* = 1.0 | *P^#^* = 0.158 |
| High MCHC | 0/11 | 0/14 | 1/13 | 0/11 | 0/13 | 0/5 | 0/10 | 0/3 | 1/4 | 1/17 | 0/10 | 0/3 |
| 1/84 | *P^#^* = 1.0 | *P^#^* = 1.0 | *P^#^* = 0.155 | *P^#^* = 1.0 | *P^#^* = 1.0 | *P^#^* = 1.0 | *P^#^* = 1.0 | *P^#^* = 1.0 | *P^#^* = 0.047* | *P^#^* = 0.202 | *P^#^* = 1.0 | *P^#^* = 1.0 |

**Additional file 6: Table S5** (continued).

| Hematological parameters ^a^ | Tests | | | | | | | | | | | |
| --- | --- | --- | --- | --- | --- | --- | --- | --- | --- | --- | --- | --- |
|  | SPLA | rK39 | rK28 | rKDDR | LicTXNPx | SPLA, rK39 and LicTXNPx positivity | Minimum of 3 positive ELISA | All ELISA positive | DAT | IFAT40 | IFAT80 | PCR |
|  | No. of cats affected by each haematological abnormality/Total no. of positive tests | | | | | | | | | | | |
| Eritrogram |  |  |  |  |  |  |  |  |  |  |  |  |
| Low MCHC | 4/11 | 3/14 | 4/13 | 3/11 | 6/13 | 2/5 | 3/10 | 2/3 | 1/4 | 6/17 | 3/10 | 0/3 |
| 34/84 | *P^#^* = 1.0 | χ^2^ = 1.670 | χ^2^ = 0.273 | *P^#^* = 0.513 | χ^2^ = 0.08 | *P^#^* = 1.0 | *P^#^* = 0.733 | *P^#^* = 0.566 | *P^#^* = 0.640 | χ^2^ = 0.044 | *P^#^* = 0.733 | *P^#^* = 0.285 |
|  |  | *P* = 0.196 | *P* = 0.547 |  | *P* = 0.928 |  |  |  |  | *P* = 0.833 |  |  |
| High MCV | 5/11 | 5/14 | 6/13 | 6/11 | 8/13 | 4/5 | 5/10 | 3/3 | 2/4 | 5/17 | 3/10 | 1/3 |
| 29/83 |  |  |  |  |  |  |  |  |  | χ^2^ = 0.063 |  |  |
|  | *P^#^* = 0.504 | *P^#^* = 1.0 | *P^#^* = 0.362 | *P^#^* = 0.177 | *P^#^* = 0.053* | *P^#^* = 0.046* | *P^#^* = 0.308 | *P^#^* = 0.038* | *P^#^* = 0.606 | *P* = 0.802 | *P^#^* = 1.0 | *P^#^* = 1.0 |
| Low MCV | 0/11 | 2/14 | 2/13 | 0/11 | 0/13 | 0/5 | 0/10 | 0/3 | 1/4 | 3/17 | 1/10 | 1/3 |
| 7/83 | *P^#^* = 0.586 | *P^#^* = 0.336 | *P^#^* = 0.301 | *P^#^* = 0.587 | *P^#^* = 0.589 | *P^#^* = 1.0 | *P^#^* = 0.590 | *P^#^* = 1.0 | *P^#^* = 0.299 | *P^#^* = 0.148 | *P^#^* = 1.0 | *P^#^* = 0.264 |
| High RDW | 1/11 | 3/14 | 4/13 | 1/10 | 2/13 | 1/5 | 1/10 | 1/3 | 2/4 | 4/16 | 3/10 | 2/3 |
| 20/82 | *P^#^* = 0.279 | *P^#^* = 1.0 | *P^#^* = 0.730 | *P^#^* = 0.275 | *P^#^* = 0.505 | *P^#^* = 1.0 | *P^#^* = *0*.438 | *P^#^* = 1.0 | *P^#^* = 0.249 | *P^#^* = 1.0 | *P^#^* = 0.700 | *P^#^* = 0.122 |
| Low RDW | 0/11 | 1/14 | 0/13 | 0/11 | 0/13 | 0/5 | 1/10 | 0/3 | 0/4 | 0/16 | 0/10 | 0/3 |
| 2/82 | *P^#^* = 1.0 | *P^#^* = 0.314 | *P^#^* = 1.0 | *P^#^* = 1.0 | *P^#^* = 1.0 | *P^#^* = 1.0 | *P^#^* = 1.0 | *P^#^* = 1.0 | *P^#^* = 1.0 | *P^#^* = 1.0 | *P^#^* = 1.0 | *P^#^* = 1.0 |
| Leukocyte count |  |  |  |  |  |  |  |  |  |  |  |  |
| Leukocytosis | 3/12 | 4/17 | 4/14 | 4/14 | 6/16 | 2/6 | 3/10 | 2/3 | 2/7 | 4/20 | 3/13 | 1/6 |
| 28/86 |  | χ^2^ = 0.358 |  |  | χ^2^ = 0.043 |  |  |  |  | χ^2^ = 1.201 |  |  |
|  | *P^#^* = 0.743 | *P =* 0.550 | *P^#^* = 0.211 | *P^#^* = 1.0 | *P* = 0.835 | *P^#^* = 1.0 | *P^#^* = 0.353 | *P^#^* = 0.241 | *P^#^* = 1.0 | *P* = 0.273 | *P^#^* = 0.533 | *P^#^* = 0.661 |
| Leukopenia | 1/12 | 0/17 | 0/14 | 0/13 | 0/16 | 0/6 | 0/10 | 0/3 | 0/7 | 2/20 | 0/13 | 1/6 |
| 5/86 | *P^#^* = 0.537 | *P^#^* = 0.578 | *P^#^* = 0.586 | *P^#^* = 1.0 | *P^#^* = 0.579 | *P^#^* = 1.0 | *P^#^* = 1.0 | *P^#^* = 1.0 | *P^#^* = 1.0 | *P^#^* = 0.329 | *P^#^* = 1.0 | *P^#^* = 0.345 |
| Neutrophilia | 1/12 | 4/13 | 3/13 | 3/11 | 4/13 | 1/5 | 3/10 | 1/3 | 0/4 | 4/17 | 3/10 | 1/3 |
| 13/80 | *P^#^* = 0.537 | *P^#^* = 0.209 | *P^#^* = 0.431 | *P^#^* = 0.371 | *P^#^* = 0.208 | *P^#^* = 1.0 | *P^#^* = 0.353 | *P^#^* = 0.413 | *P^#^* = 1.0 | *P^#^* = 0.458 | *P^#^ =* 0.353 | *P^#^* = 0.406 |
| Neutropenia | 1/12 | 0/13 | 0/13 | 0/11 | 1/13 | 0/5 | 0/10 | 0/3 | 0/4 | 3/17 | 0/10 | 1/3 |
| 12/80 | *P^#^* = 0.537 | *P^#^* = 0.199 | *P^#^* = 0.199 | *P^#^* = 0.197 | *P^#^* = 0.681 | *P^#^* = 0.587 | *P^#^* = 0.345 | *P^#^* = 1.0 | *P^#^* = 1.0 | *P^#^* = 0.710 | *P^#^* = 0.345 | *P^#^* = 0.275 |

**Additional file 6: Table S5** (continued).

| Hematological parameters ^a^ | Tests | | | | | | | | | | | |
| --- | --- | --- | --- | --- | --- | --- | --- | --- | --- | --- | --- | --- |
|  | SPLA | rK39 | rK28 | rKDDR | LicTXNPx | SPLA, rK39 and LicTXNPx positivity | Minimum of 3 positive ELISA | All ELISA positive | DAT | IFAT40 | IFAT80 | PCR |
|  | No. of cats affected by each haematological abnormality/Total no. of positive tests | | | | | | | | | | | |
| Lymphocytosis | 3/11 | 3/13 | 6/13 | 3/11 | 3/13 | 0/5 | 3/10 | 0/3 | 1/4 | 4/17 | 2/10 | 0/3 |
| 18/80 | *P^#^* = 0.704 | *P^#^* = 1.0 | *P^#^* = 0.063 | *P^#^* = 0.714 | *P^#^* = 1.0 | *P^#^* = 0.586 | *P^#^* = 0.686 | *P^#^* = 1.0 | *P^#^* = 1.0 | *P^#^* = 1.0 | *P^#^* = 1.0 | *P^#^* = 1.0 |
| Lymphopenia | 2/11 | 1/13 | 0/13 | 0/11 | 2/13 | 1/5 | 1/10 | 0/3 | 0/4 | 3/17 | 1/10 | 1/3 |
| 9/80 | *P^#^* = 0.603 | *P^#^* = 1.0 | *P^#^* = 0.342 | *P^#^* = 0.349 | *P^#^* = 0.632 | *P^#^* = 0.418 | *P^#^* = 0.688 | *P^#^* = 1.0 | *P^#^* = 1.0 | *P^#^* = 0.392 | *P^#^* = 1.0 | *P^#^* = 0.309 |
| Monocytosis | 1/11 | 1/13 | 1/13 | 1/11 | 1/13 | 1/5 | 1/10 | 1/3 | 0/4 | 0/17 | 0/10 | 1/3 |
| 6/80 | *P^#^* = 1.0 | *P^#^* = 1.0 | *P^#^* = 1.0 | *P^#^* = 1.0 | *P^#^* = 1.0 | *P^#^* = 0.326 | *P^#^* = 0.564 | *P^#^* = 0.209 | *P^#^* = 1.0 | *P^#^* = 0.333 | *P^#^* = 1.0 | *P^#^* = 0.202 |
| Eosinophilia | 0/11 | 2/13 | 5/13 | 1/11 | 2/13 | 0/5 | 1/10 | 0/3 | 1/4 | 4/17 | 2/10 | 0/3 |
| 16/80 | *P^#^* = 0.109 | *P^#^* = 1.0 | *P^#^* = 0.122 | *P^#^* = 0.684 | *P^#^* = 1.0 | *P^#^* = 0.577 | *P^#^* = 0.677 | *P^#^* = 1.0 | *P^#^* = 1.0 | *P^#^* = 0.736 | *P^#^* = 1.0 | *P^#^* = 1.0 |
| Basophilia | 0/11 | 0/13 | 0/13 | 0/11 | 0/13 | 0/5 | 0/10 | 0/3 | 0/4 | 0/17 | 0/10 | 0/3 |
| 2/80 | *P^#^ =* 1.0 | *P^#^ =* 1.0 | *P^#^ =* 1.0 | *P^#^ =* 1.0 | *P^#^ =* 1.0 | *P^#^ =* 1.0 | *P^#^ =* 1.0 | *P^#^ =* 1.0 | *P^#^ =* 1.0 | *P^#^ =* 1.0 | *P^#^* = 1.0 | *P^#^* = 1.0 |
| Platelets count |  |  |  |  |  |  |  |  |  |  |  |  |
| Thrombocytosis | 0/12 | 0/17 | 0/14 | 0/13 | 0/16 | 0/6 | 0/12 | 0/3 | 0/7 | 0/20 | 0/13 | 1/6 |
| 3/86 | *P^#^* = 1.0 | *P^#^* = 1.0 | *P^#^* = 1.0 | *P^#^* = 1.0 | *P^#^* = 1.0 | *P^#^* = 1.0 | *P^#^* = 1.0 | *P^#^* = 1.0 | *P^#^* = 1.0 | *P^#^* = 1.0 | *P^#^* = 1.0 | *P^#^* = 0.153 |
| Thrombocyto-penia | 6/12 | 6/17 | 2/14 | 4/13 | 5/16 | 4/6 | 6/12 | 1/3 | 2/7 | 7/20 | 6/13 | 2/6 |
| 15/86 | *P^#^* = 0.005* | *P^#^* = 0.067 | *P^#^* = 1.0 | *P^#^* = 0.210 | *P^#^* = 0.139 | *P^#^* = 0.007* | *P^#^* = 0.005* | *P^#^* = 0.437 | *P^#^* = 0.347 | *P^#^* = 0.038* | *P^#^* = 0.009* | *P^#^* = 0.271 |

DAT, direct agglutination test; ELISA, enzyme-linked immunosorbent assay; Hg, hemoglobin; Ht (%), hematocrit; IFAT, indirect fluorescent antibody test; LicTXNPX*, Leishmania infantum* recombinant cytosolic peroxiredoxin protein; MCHC, mean corpuscular hemoglobin concentration; rK28, *L. infantum* recombinant kinesin 28; rK39, *L. infantum* recombinant kinesin 39; RBC, red blood cell (erythrocyte) count; RDW, red blood cell distribution width; rKDDR, *L. infantum* recombinant kinesin degenerated derived repeat; SPLA, soluble promastigote *Leishmania* antigens; WBC, white blood cell (leukocyte) count.

^a^No. of abnormal haematological results/No. of cats tested.

χ^2^ and FET (^#^) computed for binomial distribution; *df* = 1 for all χ^2^ measurements.

*Statistically significant difference.
