# Supplementary material for: Diagnostic potential of a multi-antigen ELISA for feline leishmaniosis
Source: Parasit Vectors. 2026 Mar 16;19:157. doi: 10.1186/s13071-026-07320-5 (PMC13077857; doi:10.1186/s13071-026-07320-5)
Supplement: Supplementary file 7 — Additional file 7. [file 13071_2026_7320_MOESM7_ESM.docx]

**Additional file 7: Table S6** Associations between abnormal feline serum biochemistry parameters (n = 86) and a positive result obtained for ELISA antigens (SPLA, rK39, rK28, rKDDR and LicTXNPx), DAT, IFAT (40 and 80) and PCR, given by Chi-square (χ2) or Fisher´s exact test (FET).

| Serum  biochemistry parameters ^a^ | Tests | | | | | | | | | | | |
| --- | --- | --- | --- | --- | --- | --- | --- | --- | --- | --- | --- | --- |
|  | SPLA | rK39 | rK28 | rKDDR | LicTXNPx | SPLA, rK39 and LicTXNPx positivity | Minimum of 3 positive ELISA | All ELISA positive | DAT | IFAT40 | IFAT80 | PCR |
|  | No. of cats affected by each serum biochemistry abnormality/Total no. of positive tests | | | | | | | | | | | |
| High albumin | 0/12 | 0/17 | 0/14 | 0/12 | 0/16 | 0/6 | 0/12 | 0/3 | 0/7 | 0/20 | 0/13 | 0/6 |
| 2/86 | *P^#^* = 1.0 | *P^#^* = 1.0 | *P^#^* = 1.0 | *P^#^* = 1.0 | *P^#^* = 1.0 | *P^#^* = 1.0 | *P^#^* = 1.0 | *P^#^* = 1.0 | *P^#^* = 1.0 | *P^#^* = 1.0 | *P^#^* = 1.0 | *P^#^* = 1.0 |
| Low albumin | 5/12 | 8/17 | 7/14 | 4/12 | 4/16 | 4/6 | 5/12 | 3/3 | 2/7 | 8/20 | 5/13 | 3/6 |
| 29/86 |  | χ^2^ = 0.945 |  |  | χ^2^ = 0.275 |  |  |  |  | χ^2^ = 0.133 |  |  |
|  | *P^#^* = 0.528 | *P =* 0.253 | *P^#^* = 0.220 | *P^#^* = 1.0 | *P =* 0.600 | *P^#^* = 0.173 | *P^#^* = 0.528 | *P^#^* =0.036* | *P^#^* = 1.0 | *P =* 0.715 | *P^#^* = 0.756 | *P^#^* = 0.377 |
| High globulins | 6/12 | 8/17 | 7/14 | 6/12 | 7/16 | 5/6 | 7/12 | 3/3 | 3/7 | 8/20 | 6/13 | 4/6 |
| 21/85 | *P^#^* = 0.063 | *P^#^* = 0.025* | *P^#^* = 0.036* | *P^#^* = 0.064 | *P^#^* = 0.058 | *P^#^* = 0.003* | *P^#^* = 0.007* | *P^#^* = 0.013* | *P^#^* = 0.357 | *P^#^* = 0.082 | *P^#^* = 0.078 | *P^#^* = 0.033* |
| Low globulins | 0/12 | 0/17 | 0/14 | 0/12 | 0/16 | 0/6 | 0/12 | 0/3 | 0/7 | 0/20 | 0/13 | 0/6 |
| 1/85 | *P^#^* = 1.0 | *P^#^* = 1.0 | *P^#^* = 1.0 | *P^#^* = 1.0 | *P^#^* = 1.0 | *P^#^* = 1.0 | *P^#^* = 1.0 | *P^#^* = 1.0 | *P^#^* = 1.0 | *P^#^* = 1.0 | *P^#^* = 1.0 |  |
| High TP | 3/12 | 3/17 | 4/14 | 2/12 | 5/16 | 5/6 | 3/12 | 1/3 | 1/7 | 4/20 | 3/13 | 2/6 |
| 16/85 | χ^2^ = 0.283 | χ^2^ = 0.000 |  |  | χ^2^ = 2.727 |  |  |  |  | χ^2^ = 0.0 |  |  |
|  | *P =* 0.595 | *P =* 1.0 | *P^#^* = 0.453 | *P^#^* = 1.0 | *P =* 0.099 | *P^#^* = 0.309 | *P^#^* = 0.689 | *P^#^* = 0.465 | *P^#^* = 1.0 | *P =* 1.0 | *P^#^* = 0.704 | *P^#^* = 0.311 |
| Low TP | 0/12 | 1/17 | 1/14 | 1/12 | 1/16 | 0/6 | 1/12 | 0/3 | 1/7 | 2/20 | 1/13 | 1/6 |
| 5/85 | *P^#^* = 1.0 | *P^#^* = 1.0 | *P^#^* = 1.0 | *P^#^* = 0.542 | *P^#^* = 1.0 | *P^#^* = 1.0 | *P^#^* = 0.537 | *P^#^* = 1.0 | *P^#^* = 0.356 | *P^#^* = 0.587 | *P^#^* = 0.573 | *P^#^* = 0.155 |
| High A/G | 0/12 | 0/17 | 0/14 | 0/12 | 0/16 | 0/6 | 0/12 | 0/3 | 0/7 | 0/20 | 0/13 | 3/6 |
| 1/86 | *P^#^* = 1.0 | *P^#^* = 1.0 | *P^#^* = 1.0 | *P^#^* = 1.0 | *P^#^* = 1.0 | *P^#^* = 1.0 | *P^#^* = 1.0 | *P^#^* = 1.0 | *P^#^* = 1.0 | *P^#^* = 1.0 | *P^#^* = 1.0 | *P^#^* = 0.061 |
| Low A/G | 4/12 | 8/17 | 6/14 | 4/12 | 5/16 | 4/6 | 5/12 | 3/3 | 3/7 | 5/20 | 4/13 | 3/6 |
| 14/85 | *P^#^* = 0.101 | *P^#^ <* 0.001* | *P^#^* = 0.010* | *P^#^* = 0.105 | *P^#^* = 0.125 | *P^#^* = 0.006* | *P^#^* = 0.023* | *P^#^* = 0.004* | *P^#^* = 0.084 | *P^#^* = 0.302 | *P^#^* = 0.215 | *P^#^* = 0.061 |

**Additional file 7: Table S6** (continued).

| Serum  biochemistry parameters ^a^ | Tests | | | | | | | | | | | |
| --- | --- | --- | --- | --- | --- | --- | --- | --- | --- | --- | --- | --- |
|  | SPLA | rK39 | rK28 | rKDDR | LicTXNPx | SPLA, rK39 and LicTXNPx positivity | Minimum of 3 positive ELISA | All ELISA positive | DAT | IFAT40 | IFAT80 | PCR |
|  | No. of cats affected by each serum biochemistry abnormality/Total no. of positive tests | | | | | | | | | | | |
| High serum creatinine | 2/12 | 1/17 | 3/14 | 2/12 | 3/16 | 1/6 | 2/12 | 1/3 | 0/7 | 0/20 | 0/13 | 0/6 |
| 8/86 | *P^#^* = 0.309 | *P^#^* = 1.0 | *P^#^* = 0.121 | *P^#^* = 0.314 | *P^#^* = 0.169 | *P^#^* = 0.454 | *P^#^* = 0.309 | *P^#^* = 0.257 | *P^#^* = 1.0 | *P^#^* = 0.189 | *P^#^* = 0.349 | *P^#^* = 1.0 |
| High BUN | 3/12 | 4/17 | 6/14 | 3/12 | 4/16 | 2/6 | 2/12 | 2/3 | 2/7 | 5/19 | 3/13 | 0/6 |
| 20/85 | *P^#^* = 1.0 | *P^#^* = 1.0 | *P^#^* = 0.076 | *P^#^* = 1.0 | *P^#^* = 1.0 | *P^#^* = 0.622 | *P^#^* = 0.723 | *P^#^* = 0.137 | *P^#^* = 0.654 | *P^#^* = 0.757 | *P^#^* = 1.0 | *P^#^* = 0.581 |
| High ALT | 2/12 | 3/17 | 4/14 | 3/12 | 3/16 | 0/6 | 2/12 | 0/3 | 2/7 | 3/20 | 2/13 | 1/6 |
| 29/86 |  | χ^2^ = 1.635 |  |  | χ^2^ = 1.234 |  |  |  |  | χ^2^ = 3.213 |  |  |
|  | *P^#^* = 0.323 | *P =* 0.201 | *P^#^* = 0.763 | *P^#^* = 0.744 | *P =* 0.267 | *P^#^* = 0.093 | *P^#^* = 0.323 | *P^#^* = 0.548 | *P^#^* = 1.0 | *P =* 0.073 | *P^#^* = 0.203 | *P^#^* = 0.658 |
| High ALK | 1/11 | 0/14 | 0/13 | 1/10 | 0/13 | 0/5 | 0/10 | 0/2 | 0/4 | 0/17 | 0/10 | 1/3 |
| 4/83 | *P^#^* = 0.440 | *P^#^* = 1.0 | *P^#^* = 1.0 | *P^#^* = 0.412 | *P^#^* = 1.0 | *P^#^* = 1.0 | *P^#^* = 1.0 | *P^#^* = 1.0 | *P^#^* = 1.0 | *P^#^* = 0.575 | *P^#^* = 1.0 | *P^#^* = 0.082 |

Alb, albumin; ALT, alanine aminotransferase; A/G, albumin/ globulin ratio; ALK, alkaline phosphatase; BUN, blood urea nitrogen; DAT, direct agglutination test; ELISA, enzyme-linked immunosorbent assay; IFAT, indirect fluorescent antibody test; LicTXNPx*, Leishmania infantum* recombinant cytosolic peroxiredoxin protein; PCR, polymerase chain reaction; rK28, *L. infantum* recombinant kinesin 28; rK39, *L. infantum* recombinant kinesin 39; rKDDR, *L. infantum* recombinant kinesin degenerated derived repeat; SPLA, soluble promastigote *Leishmania* antigens; TP, total proteins.

^a^No. of abnormal serum biochemistry results/No. of cats tested.

χ^2^ and FET (^#^) computed for binomial distribution; *df* = 1 for all χ^2^ measurements.

*Statistically significant difference.
