# Supplementary material for: Diagnostic potential of a multi-antigen ELISA for feline leishmaniosis
Source: Parasit Vectors. 2026 Mar 16;19:157. doi: 10.1186/s13071-026-07320-5 (PMC13077857; doi:10.1186/s13071-026-07320-5)
Supplement: Supplementary file 8 — Additional file 8. [file 13071_2026_7320_MOESM8_ESM.docx]

**Additional file 8: Table S7** Associations between feline signalment (age [< 3 years, n = 163; > 3 years, n = 49], sex [male, n = 97; female, n = 115]) and positivity to ELISA antigens (SPLA, rK39, rK28, rKDDR and LicTXNPx), DAT, IFAT and PCR given by Chi-square (χ2) or Fisher´s exact test (FET).

| Signalment | Tests |  |  |  |  |  |  |  |  |  |  |  |
| --- | --- | --- | --- | --- | --- | --- | --- | --- | --- | --- | --- | --- |
|  | SPLA | rK39 | rK28 | rKDDR | LicTXNPx | SPLA, rK39 and LicTXNPx positivity | Minimum of 3 positive ELISA | All ELISA positive | DAT | IFAT 40 | IFAT 80 | PCR |
|  | No. of positives for each test and percentage (%) of positivity | | | | | | | | | | | |
| Age |  |  |  |  |  |  |  |  |  |  |  |  |
| < 3 years | 23 (14.1) | 18 (11.0) | 19 (11.7) | 17 (10.4) | 24 (14.7) | 7 (4.3) | 16 (9.8) | 3 (1.8) | 8 (4.9) | 24 (14.7) | 13 (8.0) | 1 (0.6) |
|  |  |  |  |  |  |  |  |  |  |  |  |  |
| > 3 years | 12 (24.5) | 14 (28.6) | 13 (26.5) | 12 (24.5) | 11 (22.4) | 10 (20.4) | 12 (24.5) | 2 (4.1) | 6 (12.2) | 11 (22.4) | 7 (14.3) | 5 (10.2) |
|  |  |  |  |  |  |  |  |  |  |  |  |  |
|  | χ^2^ = 5.840 | χ^2^ = 13.195 | χ^2^ = 10.723 | χ^2^ = 10.114 | χ^2^ =3.760 | χ^2^ = 17.30 | χ^2^ = 11.049 | *P* = 0.229 | *P =* 0.026* | χ^2^ = 3.760 | *P =* 0.064 | *P <* 0.001* |
|  | *P =* 0.016* | *P =* 0.002* | *P* < 0.001* | *P =* 0.001* | *P =* 0.052* | *P* < 0.001* | *P* < 0.001* |  |  | *P* = 0.052* |  |  |
| Sex |  |  |  |  |  |  |  |  |  |  |  |  |
| Male | 22 (13.5) | 22 (13.5) | 24 (14.7) | 19 ((11.7) | 23 (14.1) | 12 (7.4) | 21 (12.9) | 4 (2.5) | 8 (4.9) | 17 (10.4) | 12 (7.4) | 4 (2.5) |
| 97/228 |  |  |  |  |  |  |  |  |  |  |  |  |
| Female | 13 (26.5) | 11 (22.4) | 8 (16.3) | 10 (20.4) | 12 (24.5) | 5 (10.2) | 7 (14.3) | 1 (2.0) | 6 (12.2) | 18 (36.7) | 8 (16.3) | 2 (4.1) |
| 13/115 |  |  |  |  |  |  |  |  |  |  |  |  |
|  | χ^2^ 4.149 | χ^2^ 5.925 | χ^2^ 11.637 | χ^2^ 4.404 | χ^2^ 5.800 | χ^2^ 3.569 | χ^2^ 9.801 | χ^2^ 1.213 | χ^2^ 0.369 | χ^2^ 0.033 | χ^2^ 1.227 |  |
|  | *P =* 0.042* | *P =* 0.015* | *P<*0.001* | *P =* 0.036* | *P =* 0.016* | *P =* 0.059 | *P =* 0.002* | *P =* 0.271 | *P =* 0.544 | *P =* 0.857 | *P =* 0.268 | *P =* 0.426 |

DAT, direct agglutination test; ELISA, enzyme-linked immunosorbent assay; IFAT, indirect fluorescence antibody test; LicTXNPx*, L. infantum* recombinant cytosolic peroxiredoxin protein; PCR, polymerase chain reaction; rK28, *L. infantum* recombinant kinesin 28; rK39, *L. infantum* recombinant kinesin 39; rKDDR, *L. infantum* recombinant kinesin degenerated derived repeat; SPLA, soluble promastigote *Leishmania* antigens.

χ^2^ and FET computed for binomial distribution; *df* = 1 for all χ^2^ measurements.

*Statistically significant difference.
